# Lasting consequences of psyllid (*Bactericera cockerelli* L.) infestation on tomato defense, gene expression, and growth

**DOI:** 10.1186/s12870-021-02876-z

**Published:** 2021-02-24

**Authors:** Kyle Harrison, Azucena Mendoza-Herrera, Julien Gad Levy, Cecilia Tamborindeguy

**Affiliations:** 1 USDA-ARS, Agroecosystem Management Research Unit, Lincoln, NE 68503 USA; 2 Department of Entomology, Texas A&M University, College Station, TX 77843 USA; 3 Department of Horticultural Sciences, Texas A&M University, College Station, TX 77843 USA

**Keywords:** *Bactericera cockerelli* Šulc, *Solanum lycopersicum* L., Transcriptomics, Plant-insect interactions, Potato, Psyllid, Zebra chip, ‘*Candidatus* Liberibacter solanacearum’

## Abstract

**Background:**

The tomato psyllid, *Bactericera cockerelli* Šulc (Hemiptera: Triozidae), is a pest of solanaceous crops such as tomato (*Solanum lycopersicum* L.) in the U.S. and vectors the disease-causing pathogen ‘*Candidatus* Liberibacter solanacearum’. Currently, the only effective strategies for controlling the diseases associated with this pathogen involve regular pesticide applications to manage psyllid population density. However, such practices are unsustainable and will eventually lead to widespread pesticide resistance in psyllids. Therefore, new control strategies must be developed to increase host-plant resistance to insect vectors. For example, expression of constitutive and inducible plant defenses can be improved through selection. Currently, it is still unknown whether psyllid infestation has any lasting consequences on tomato plant defense or tomato plant gene expression in general.

**Results:**

In order to characterize the genes putatively involved in tomato defense against psyllid infestation, RNA was extracted from psyllid-infested and uninfested tomato leaves (Moneymaker) 3 weeks post-infestation. Transcriptome analysis identified 362 differentially expressed genes. These differentially expressed genes were primarily associated with defense responses to abiotic/biotic stress, transcription/translation, cellular signaling/transport, and photosynthesis. These gene expression changes suggested that tomato plants underwent a reduction in plant growth/health in exchange for improved defense against stress that was observable 3 weeks after psyllid infestation. Consistent with these observations, tomato plant growth experiments determined that the plants were shorter 3 weeks after psyllid infestation. Furthermore, psyllid nymphs had lower survival rates on tomato plants that had been previously psyllid infested.

**Conclusion:**

These results suggested that psyllid infestation has lasting consequences for tomato gene expression, defense, and growth.

**Supplementary Information:**

The online version contains supplementary material available at 10.1186/s12870-021-02876-z.

## Background

The tomato psyllid (or potato psyllid), *Bactericera cockerelli* Šulc (Hemiptera: Triozidae), is a major pest of solanaceous crops such as tomato (*Solanum lycopersicum* L.) and potato (*S. tuberosum*) in the U.S. [8]. The psyllid is native to the Southwestern U.S. and Northern Mexico [12, 49, 55, 64] but has only recently become an important agricultural pest when it was discovered that *B. cockerelli* vectors the disease-causing pathogen ‘*Candidatus* Liberibacter solanacearum’ (Lso) [43]. Lso is a fastidious bacterial pathogen associated with zebra chip disease in potato as well as other diseases in solanaceous crops [37, 41]. Today, Lso is considered a major pathogen of crops worldwide [20, 63]. Currently, the only effective strategies for controlling the diseases associated with Lso involve calendar application of insecticide [8, 42]. However, these strategies are unsustainable. Multiple reports indicate neonicotinoid resistance is increasing in certain *B. cockerelli* populations [5, 45, 50]. Since vector-borne disease systems are faced with the rapid evolution of pesticide resistance, major efforts have been made to develop novel solutions based on selectively breeding plants for improved host-plant resistance or genetically manipulating plants and insects for the purpose of disrupting disease transmission [3, 4, 34, 35, 57, 66]. For example, disease transmission can be disrupted by manipulating the host or vector’s genes associated with key molecular pathways that facilitate the movement of pathogens from host to vector and vice versa [1, 32]. Such genetic manipulations can be accomplished through direct transformations or artificial selection, but these toolkits require certain a priori genomic information. Therefore, in order to pursue psyllid control strategies that manipulate the host plant’s molecular pathways, the current study identifies the genes involved in the transcriptomic response of tomato plants to psyllid infestation.

The current study focuses on an insect-plant relationship, however the experiments described are informed by Lso disease development. Specifically, diseases caused by Lso are characterized by long latent periods. Indeed, symptoms in tomato and potato typically start developing 3 weeks after infection [33, 40, 43, 59]. Logically, studies of Lso infection are conducted a few weeks or even months after plants are infested with psyllids and subsequently infected with Lso. To avoid the confounding effects of psyllid herbivory, some studies entirely divorce the effect of vector infestation by transmitting the pathogen from one host-plant to another via grafting [13, 59]. Furthermore, the rate of Lso infection and disease development are independent of psyllid density [52]. Thus, the long-term effects of psyllid infestation on tomato plant biology and gene expression are divorced from Lso research and are still unknown. This is important knowledge gap considering psyllids are known to cause phenotypic changes in solanaceous crops under heavy infestation (≥100 insects per plant), a condition called ‘psyllid yellows’ [7, 60]. Typically, studies of Lso infection have involved a single control group of plants that have not been exposed to either the psyllid vector or the Lso pathogen. Then, controls will be compared to plants exposed to both the psyllid and Lso. This practice has been acceptable because psyllid-responsive expression changes in plants are expected to be relatively unimportant compared to Lso challenge. Although this experimental design has been invaluable for characterizing Lso disease severity and psyllid transmission efficacy, an unintended consequence is the knowledge gap regarding the lasting consequences of psyllid infestation on tomato plant health. The molecular interaction between host plant and insect vector is especially important because plants have several long-term responses to insect damage that can impact their lifetime health, reproduction, and defense.

Plants undergo physiological, transcriptomic, or epigenetic changes which allows them to mount a stronger and faster responses to secondary challenges by previously perceived threats. This is called defense ‘priming’ [10, 21, 30, 39]. Priming is a common phenomenon that has been studied in several plant species in response to bacteria, fungi, and chewing insects [11, 24, 61, 68]. Furthermore, plants can remain immunologically primed for the rest of their lives or even across generations [47, 53, 62]. Therefore, it is reasonable to hypothesize that tomato plants deploy similar long-term defenses against psyllids post-infestation and that these changes have lasting consequences for tomato survival, growth, and development. In fact, the lasting the consequences of uninfected psyllid infestation were previously observed (but not quantified) in a study by Mendoza Herrera et al. [40].

The current study evaluated the persistent transcriptomic and physical responses of tomato plants to psyllid infestation. This was accomplished by comparing the transcriptomes of uninfested plants to plants that had been infested 3 weeks prior. Second, tomato plant growth was tracked across time to test the relationship between plant growth/development and immune response to psyllid infestation. This experimental design allows for the identification of genes involved in the tomato plant’s response to psyllid infestation and whether these genes were associated with improved defense against psyllids. Third, psyllid populations were monitored for the number of eggs laid and nymphal survival when reared on previously uninfested tomato plants (controls) compared to psyllids reared on previously infested plants.

## Results

### 1-Transcriptomic analysis

**Fig. 1:**
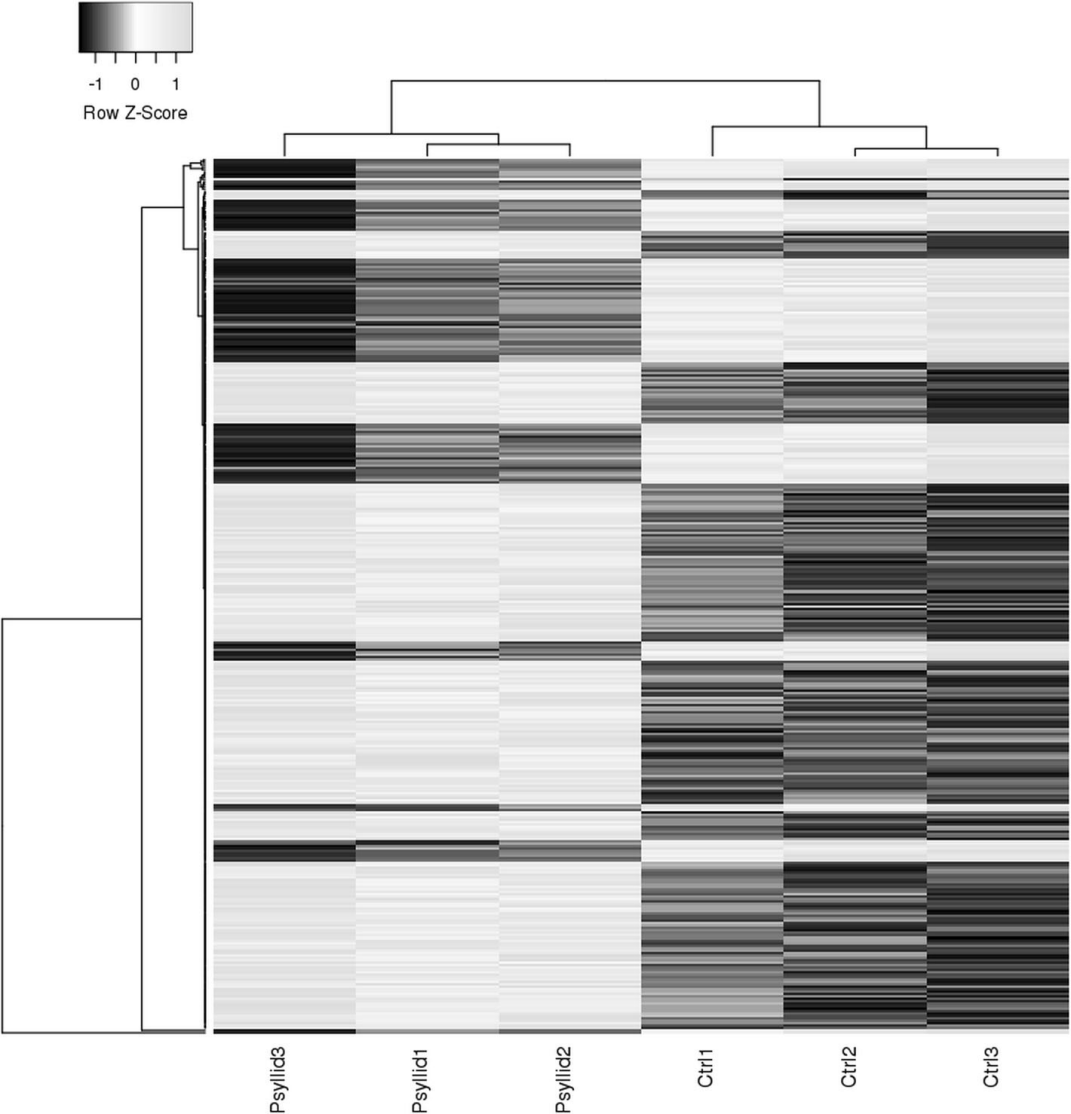
Comparative heatmap of relative expression changes among psyllid-infested (Psyllid#) and uninfested (Ctrl#) tomato plant DEGs. Dark colors denote down-regulation and light colors denote up-regulation. Lines above and to the left of the heatmap depict the phylogenetic hiearchy among similar treatments and similar gene expression levels

**Fig. 2:**
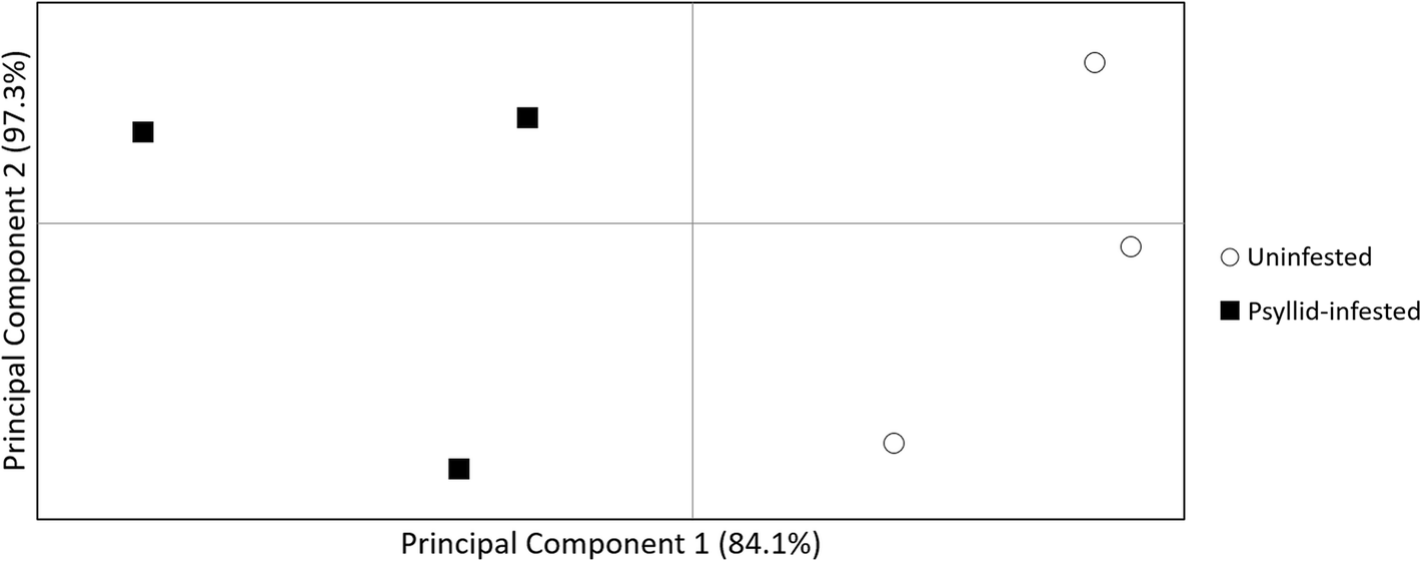
Principal component analysis (PCA) of fragments per kilobase per million reads (fpkm) among treatments psyllid-infested and uninfested tomato plants. Percentages depict the cumulative percent of the total variance explained by the associated principal axis

Illumina sequencing of tomato cDNA libraries produced 95.2 million reads that met FastQC quality control criteria (i.e., Phred quality scores > 35). The average number of reads obtained from uninfested plants (17.4 ± 0.6 million) did not significantly differ from psyllid-infested ones (18.0 ± 0.4 million) (t-value = − 0.68; *P* = 0.25). HISAT2 alignment analysis showed that 96.3 ± 0.1% of all reads from uninfested plants and 96.2 ± 0.3% of all reads from psyllid-infested plants mapped to vSL3.0 of the *S. lycopersicum* genome (Supplementary Table 2); these alignment rates did not significantly differ (t-value = 0.14; *P* = 0.45). The Ballgown analysis identified 362 differentially expressed genes (DEGs) between control and psyllid-infested plants (q-value < 0.01). These DEGs represented the pattern of systemic tomato plant gene expression following psyllid infestation. Gene expression patterns were visualized with a heatmap comparing the fold change (Z-Score) for each gene between samples (Fig. 1); Z-scores based on deviations from the average fpkm (fragments per kilobase per million read) value for a given gene. Additionally, a dendrogram (Fig. 1) and a principal component analysis (PCA, Fig. 2) comparing fpkm values across genes and samples were used to visualize relative similarities in gene expression across samples. Both the dendrogram and the PCA geometries suggested that the overall pattern of gene expression was consistent within each treatment, where per-gene fpkm values were most similar within treatment and most different between treatments. Furthermore, the PCA showed that the first principal component strongly separated the fpkm values of psyllid-infested plants from uninfested plants and accounted for 84.1% of the total variance in fpkm values, meaning the greatest differences in gene expression between samples were the differences between infested and uninfested plants.

**Fig. 3:**
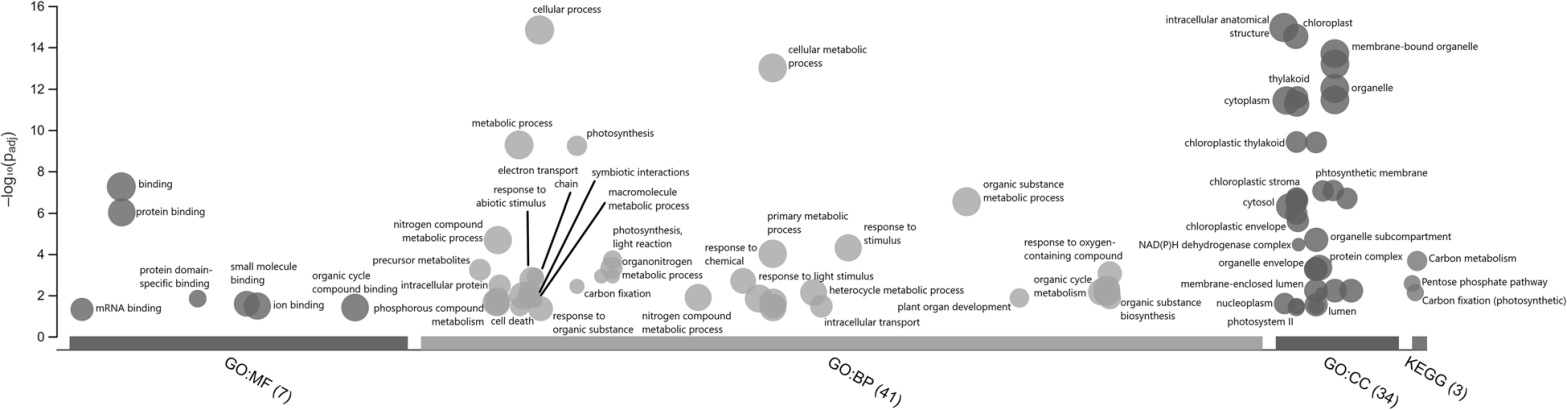
g:Profiler analysis of tomato plant DEG homologs depicting their relative overrepresentation among *Arabidopsis* molecular functions (MF), biological processes (BP), or cellular components (CC). The left axis represents the -log_10_(p_adj_) likelihood that a given MF, BP, or CC would be associated with a random selection of *Arabidopsis* genes. Circle sizes represent the relative number of times a given MF, BP, or CC appears among analyzed genes. In general, expression changes occurred throughout the cell and were most likely to be involved with cellular processes, metabolism, photosynthesis, response to stimulus, and biological regulation. Labels above, connected to arrows, or adjacent to circles describe specific the MF, BP, or CC associated with each circle; some labels have been omitted due to redundancy

Among the 362 DEGs, 246 (67.9%) were up-regulated in psyllid-infested plants. In addition, 226 (62.4%) DEGs could be assigned a putative function based on the previously published functional analyses of tomato genes or the functional analyses of tomato gene homologs in different model organisms such as *Arabidopsis thaliana,* corn, potato, rice, or tobacco. The g:Profiler analysis (https://biit.cs.ut.ee/gplink/l/iZL80ldPRt) showed 251 DEGs (69.3%) could be assigned to two or more GO functional categories (Fig. 3; See Supplementary Figure 3 for details). Tomato plant DEGs were assigned to one or more of the following broader categories: Defense response to biotic or abiotic stress (55 DEGs), transcription/translation (50 DEGs), photosynthesis (35 DEGs), molecular signaling (33 DEGs), molecular transport (31 DEGs), reproduction (27 DEGs), protein phosphorylation/ubiquitination (26 DEGs), cellular turnover (23 DEGs), sugar metabolism (20 DEGs), ion transport/homeostasis (16 DEGs), auxin signaling (9 DEGs), and cell wall biosynthesis/metabolism (6 DEGs) (Tables 1, 2, 3 and 4). RT-qPCR corroborated the relative expression levels in tested genes: Results showed that the unchanged PIP2–4 (Solyc06g011350.2) was expressed at similar levels in both uninfested (1.13 ± 0.01) and psyllid-infested plants (1.12 ± 0.01; t-value = 0.69, *P* = 0.26). The upregulated DRIP2 (Solyc06g084040.2) was expressed at significantly lower level in control (1.15 ± 0.02) compared to psyllid infested (1.36 ± 0.03; t-value = − 6.54, *P* < 0.01). The downregulated LON2 (Solyc04g080860.1) was expressed at significantly higher levels in control (1.45 ± 0.11) compared to psyllid infested (1.01 ± 0.06; t-value = 4.04, *P* < 0.01). Lastly, the downregulated D27 (Solyc08g008630.2) was expressed at significantly higher levels in control (1.26 ± 0.08) compared to psyllid infested (0.83 ± 0.08; t-value = 4.10, *P* < 0.01).

**Table 1:** The 55 tomato plant DEGs associated with defense response to abiotic and biotic stress. DEGs were sorted by log2-fold change (log2FC). These DEGs were identified in the transcriptome analysis comparing psyllid-infested and uninfested tomato plants 3 weeks after infestation (*P* < 0.01). NCBI Blast searches were used to identify Gene IDs and protein products in tomatoes as well as their homologs in other species. Specifically, the expression changes in 44 genes (80%, **in bold**) would have resulted in improvements to plant defense pathways. These DEGs were related to defense against insect damage, microbial infection, programmed cell death, salt stress, and drought. Simultaneously, 11 DEGs, especially those related to the hypersensitive response, underwent expression changes that would have resulted in impairments to plant defense pathways

Tomato gene ID	Gene ID	Homolog	Log2FC	Protein name	Uniprot description	Effect of psyllid infestation	Citation
Solyc12g055920.1	CBL4	AT5G24270	-1.33	calcineurin B protein 4	Calcium sensor that regulates intracellular Na+ and K+ homeostasis and salt tolerance; Activates the plasma membrane Na+/H+ antiporter SOS1	Decreased potassium and calcium stress response; Decreased hypotonic salinity response	Halfter, Ursula, et al. "The Arabidopsis SOS2 protein kinase physically interacts with and is activated by the calcium-binding protein SOS3." Proceedings of the National Academy of Sciences 97.7 (2000): 3735-3740.
Solyc10g076700.1	STR10	AT3G08920	-0.90	rhodanese domain-containing protein 10	Involved in response to cold stress	Decreased reponse to cold stress	Bauer, Michael, and Jutta Papenbrock. "Identification and characterization of single-domain thiosulfate sulfurtransferases from Arabidopsis thaliana." FEBS letters 532.3 (2002): 427-431.
**Solyc06g073260.2**	**CSP41B**	**AT1G09340**	**-0.81**	**chloroplast stem-loop binding protein of 41 kDa b, chloroplastic**	Associates with pre-ribosomal particles in chloroplasts and participates in chloroplast ribosomal RNA metabolism; Required for chloroplast integrity and embryo development; Regulates the circadian system; Regulates heteroglycans and monosaccharide mobilization	Impaired chloroplast organization; Impaired circadian rhythm; Decreased defense response to bacteria and wounding; Decreased galactose catabolism; Decreased monosaccharide metabolism; Decreased transcription and translation; Decreased response to cold and drought; Decreased rRNA processing	Raab, Sabine, et al. "ABA-responsive RNA-binding proteins are involved in chloroplast and stromule function in Arabidopsis seedlings." Planta 224.4 (2006): 900-914.
**Solyc01g103760.2**	**N/a**	**AT1G71900**	**-0.71**	**magnesium transporter NIPA4**	Divalent cation transporter; Negative regulator of antiviral defense response	Decreased magnesium ion transport; Increased antiviral defense response	Gao, Hua, et al. "Arabidopsis ENOR3 regulates RNAi-mediated antiviral defense." Journal of Genetics and Genomics 45.1 (2018): 33-40.
Solyc02g093230.2	CCOAOMT1	AT4G34050	-0.69	caffeoyl-CoA O-methyltransferase	Methylates caffeoyl-CoA to feruloyl-CoA and 5-hydroxyferuloyl-CoA; Plays a role in the synthesis of feruloylated polysaccharides; Reinforces the plant cell wall; Regulates response to wounding or pathogen challenge	Decreased lignin biosynthesis; Decreased response to wounding or pathogen challenge	Civardi, L., J. Rigau, and P. Puigdomenech. "Nucleotide Sequence of two cDNAs coding for Caffeoyl-coenzyme A O-Methyltransferase (CCoAOMT) and study of their expression in Zea mays." Plant Physiol 120.4 (1999): 1.
Solyc07g032640.1	PSBO1	AT5G66570	-0.66	oxygen-evolving enhancer protein 1-1, chloroplastic	Stabilizes the manganese cluster which is the primary site of water splitting	Decreased defense response to bacteria; Decreased photoinhibition; Decreased photosynthesis; Decreased photosystem II assembly and stabilization; Decreased regulation of protein dephosphorylation	Murakami, Reiko, et al. "Characterization of an Arabidopsis thaliana mutant with impaired psbO, one of two genes encoding extrinsic 33-kDa proteins in photosystem II." FEBS letters 523.1-3 (2002): 138-142.
**Solyc08g076220.2**	**N/a**	**AT1G32060**	**-0.64**	**phosphoribulokinase, chloroplastic**	Involved in reductive pentose-phosphate cycle; Involved in defense response to bacteria and cold stress	Decreased defense response to bacteria; Impaired reductive pentose-phosphate cycle; Decreased response to cold stress	Kiddle, Guy, et al. "Effects of leaf ascorbate content on defense and photosynthesis gene expression in Arabidopsis thaliana." Antioxidants and Redox Signaling 5.1 (2003): 23-32.
Solyc02g091560.2	SHM1	AT4G37930	-0.53	serine hydroxymethyltransferase 1, mitochondrial	Catalyzes interconversion of serine and glycine in the photorespiratory pathway; Involved in controlling cell damage caused by abiotic stress; Regulates the hypersensitive defense response	Decreased response to tetrahydrofolate; Decreased L-serine metabolism; Decreased one-carbon metabolism; Decreased photorespiration; Decreased hypersensitive response; Decreased response to cadmium, cold, heat, and light stress; Decreased tetrahydrofolate metabolism	Moreno, Juan Ignacio, et al. "Arabidopsis SHMT1, a serine hydroxymethyltransferase that functions in the photorespiratory pathway influences resistance to biotic and abiotic stress." The Plant Journal 41.3 (2005): 451-463.
Solyc01g107660.2	SEP1	AT4G34190	-0.47	stress enhanced protein 1 protein	Involved in non-photochemical quenching; Plays a role in the thylakoid membrane in response to light stress	Decreased response to high light intensity; Decreased photosynthesis; Decreased response to wounding	Maejima, Kensaku, et al. "Degradation of class E MADS-domain transcription factors in Arabidopsis by a phytoplasmal effector, phyllogen." Plant signaling & behavior 10.8 (2015): e1042635.
Solyc05g008370.1	RPI2	AT2G01290	-0.38	ribose-5-phosphate isomerase 2	Catalyzes the reversible conversion of ribose-5-phosphate to ribulose 5-phosphate	Decreased prorammed cell death; Decreased pentose-phosphate shunt, non-oxidative branch; Decreased vegetative-to-reproductive phase transition of meristem; Decreased hypersensitive response	Xiong, Yuqing, et al. "Deficiency in a cytosolic ribose-5-phosphate isomerase causes chloroplast dysfunction, late flowering and premature cell death in Arabidopsis." Physiologia plantarum 137.3 (2009): 249-263.
Solyc05g006990.2	NPF4.6	AT1G69850	-0.29	protein NRT1/ PTR FAMILY 4.6	Low-affinity proton-dependent nitrate transporter; Involved in constitutive nitrate uptake; Involved in (+)-abscisic acid (ABA) transport; Mediates cellular ABA uptake	Decreased abscisic acid transport; Decreased nitrate assimilation; Decreased regulation of stomatal movement; Decreased response to nematode	Huang, Nien-Chen, et al. "Cloning and functional characterization of an Arabidopsis nitrate transporter gene that encodes a constitutive component of low-affinity uptake." The Plant Cell 11.8 (1999): 1381-1392.
**Solyc01g094680.2**	**SPPL2**	**AT1G63690**	**0.25**	**signal peptide peptidase-like 2**	Involved in pathogen defense response	Increased defense response to pathogens	Ascencio-Ibáñez, José Trinidad, et al. "Global analysis of Arabidopsis gene expression uncovers a complex array of changes impacting pathogen response and cell cycle during geminivirus infection." Plant physiology 148.1 (2008): 436-454.
**Solyc03g034200.2**	**RCF3**	**AT5G53060**	**0.26**	**RNA-binding KH domain-containing protein RCF3**	Negative regulator of osmotic stress-induced gene expression; Regulates thermotolerance responses under heat stress; Forms a complex with CPL1 that modulates co-transcriptional processes; Represses stress-inducible gene expression; Involved in primary miRNA processing and pre-miRNA biogenesis; Involved in JA-mediated fungal defense	Increased heat acclimation; Increased jasmonic acid-mediated signaling; Increased mRNA processing; Increased miRNA processing; Increased regulation of defense response to fungus; Increased regulation of gene expression; Increased response to osmotic stress; Increased RNA splicing	Xiong, Liming, et al. "HOS5–a negative regulator of osmotic stress-induced gene expression in Arabidopsis thaliana." The Plant Journal 19.5 (1999): 569-578.
**Solyc04g056280.2**	**CDKC-1**	**AT5G10270**	**0.26**	**cyclin dependent kinase C-1**	Postranscriptional modifier; Involved in protein phosphorylation; Involved in leaf growth and development; Involved in defense reponse to virus	Increased leaf development; Increased phosphorylation of RNA polymerase II C-terminal domain; Increased defense response to virus	Pischke, Melissa S., et al. "A transcriptome-based characterization of habituation in plant tissue culture." Plant Physiology 140.4 (2006): 1255-1278.
**Solyc05g048850.2**	**RH8**	**AT4G00660**	**0.27**	**DEAD-box ATP-dependent RNA helicase 8**	ATP-dependent RNA helicase involved in mRNA turnover and mRNA decapping	Increased cytoplasmic mRNA processing body assembly and mRNA transport; Increased regulation of translation; Increased stress granule assembly; Increased viral process	Baek, Woonhee, et al. "A DEAD-box RNA helicase, RH8, is critical for regulation of ABA signalling and the drought stress response via inhibition of PP2CA activity." Plant, cell & environment 41.7 (2018): 1593-1604.
**Solyc06g008970.2**	**XPD**	**AT1G03190**	**0.28**	**general transcription and DNA repair factor IIH helicase subunit XPD**	Component of the general transcription and DNA repair factor IIH core comple; Plays an essential role in transcription initiation; Essential during plant growth; Negatively regulates a response to UV damage and heat stress	Increased DNA repair; Increased mitotic recombination; Increased transcription; Increased protein phosphorylation; Increased regulation of mitotic recombination; Increased response to heat, oxidative, and UV stress; Increased transcription by RNA polymerase II	Liu, Zongrang, et al. "Arabidopsis UVH6, a homolog of human XPD and yeast RAD3 DNA repair genes, functions in DNA repair and is essential for plant growth." Plant physiology 132.3 (2003): 1405-1414.
**Solyc01g096290.2**	**RPL40A**	**AT2G36170**	**0.28**	**ubiquitin-60S ribosomal protein L40**	Involved in protein degradation via the proteasome; Linear polymer chains formed via attachment by the initiator Met-lead during cellular signaling	Increased modification-dependent protein catabolism; Increased protein ubiquitination; Increased translation; Increased defense response to bacteria	Ditt, Renata F., et al. "The Arabidopsis thaliana transcriptome in response to Agrobacteria tumefaciens." Molecular plant-microbe interactions 19.6 (2006): 665-681.
**Solyc04g082560.2**	**ITSN2**	**N/a**	**0.29**	**intersectin-2**	Adapter protein that provides indirect link between the endocytic membrane and the actin assembly machinery; Regulates the formation of clathrin-coated vesicles; Involved in endocytosis of integrin beta-1 and transferrin receptor	Increased endocytosis; Increased dendrite extension; Increased regulation of Rho protein signal transduction; Increased viral process	Mettlen, Marcel, et al. "Endocytic accessory proteins are functionally distinguished by their differential effects on the maturation of clathrin-coated pits." Molecular biology of the cell 20.14 (2009): 3251-3260.
**Solyc06g062350.2**	**RIN1**	**AT5G22330**	**0.29**	**ruvB-like protein 1**	Core component of the chromatin remodeling INO80 complex which is involved in transcriptional regulation, DNA replication, and DNA repair; Component of the NuA4 histone acetyltransferase complex involved in transcriptional activation of select genes	Increased box C/D snoRNP assembly; Increased cell differentiation; Increased chromatin remodeling; Increased flower development; Increased meristem development; Increased regulation of defense response to fungus; Increased regulation of transcription by RNA polymerase II	Heyndrickx, Ken S., and Klaas Vandepoele. "Systematic identification of functional plant modules through the integration of complementary data sources." Plant physiology 159.3 (2012): 884-901.
**Solyc11g005130.1**	**UBN1**	**AT1G21610**	**0.30**	**ubinuclein-1**	Required for replication-independent chromatin assembly	Increased nucleosome organization; Increased regulation of gene silencing; Increased response to salt stress	Nie, Xin, et al. "The HIRA complex that deposits the histone H3. 3 is conserved in Arabidopsis and facilitates transcriptional dynamics." Biology open 3.9 (2014): 794-802.
**Solyc02g079040.2**	**CBP60B**	**AT5G57580**	**0.30**	**calmodulin-binding protein 60 B**	Transcription activator that binds DNA in a sequence-specific manner to promote the expression of target genes	Increased salicylic acid biosynthesis; Increased defense response to bacteria	Reddy, Vaka S., et al. "Genes encoding calmodulin-binding proteins in the Arabidopsis genome." Journal of Biological Chemistry 277.12 (2002): 9840-9852.
**Solyc10g044910.1**	**N/a**	**AT4G06676**	**0.31**	**protein EI24 homolog**	Regulator of macroautophagy	Increased macroautophagy; Increased programmed cell death	Cheng, Chia-Yi, et al. "Araport11: a complete reannotation of the Arabidopsis thaliana reference genome." The Plant Journal 89.4 (2017): 789-804.
Solyc01g104970.2	**BAK1**	**AT4G33430**	**0.31**	**brassinosteroid insensitive 1-associated receptor kinase 1**	Involved in brassinosteroid signaling response to beacterium/fungi/oomycetes; Mediates programmed cell death	Increased brassinosteroid mediated signaling pathway; Increased programmed cell death; Increased defense response to bacteria/fungus/oomycetes	Li, Jia, et al. "BAK1, an Arabidopsis LRR receptor-like protein kinase, interacts with BRI1 and modulates brassinosteroid signaling." Cell 110.2 (2002): 213-222.
**Solyc10g083610.1**	**CTR1**	**AT5G03730**	**0.31**	**serine/threonine-protein kinase CTR1**	Ethylene receptor related to bacterial two-component regulators; Acts as a redundant negative regulator of ethylene signaling;	Increased cellular turnover; Increased cytokinin metabolism; Increased defense response to bacteria and insect damage; Increased response to ethylene; Increased hydrogen peroxide biosynthesis; Increased phloem/xylem histogenesis; Increased regulation of seedling development; Increased regulation of stomatal opening; Increased response to abscisic acid, auxin, and gibberellin; Increased response to heat and salt stress	Chang, Caren, et al. "Arabidopsis ethylene-response gene ETR1: similarity of product to two-component regulators." Science 262.5133 (1993): 539-544.
**Solyc11g013260.1**	**PHB3**	**AT5G40770**	**0.32**	**prohibitin-3, mitochondrial**	Holdase/unfoldase involved in the stabilization of newly synthesized mitochondrial proteins; Necessary for mitochondrial and cell metabolism and biogenesis; Required to regulate ethylene-mediated signaling; Involved in growth maintenance; Functions in nitric oxide-mediated responses	Increased cellular turnover; Increased defense response to bacteria; Increased lateral root development; Increased mitochondrion organization; Increased response to auxin, ethylene, and nitric oxide; Increased to salt stress; Increased salicylic acid biosynthesis	Christians, Matthew J., and Paul B. Larsen. "Mutational loss of the prohibitin AtPHB3 results in an extreme constitutive ethylene response phenotype coupled with partial loss of ethylene-inducible gene expression in Arabidopsis seedlings." Journal of experimental botany 58.8 (2007): 2237-2248.
**Solyc08g059660.1**	**SEU**	**AT1G43850**	**0.32**	**transcriptional corepressor SEUSS**	DNA-binding adapter subunit of the SEU-LUG transcriptional corepressor of AGAMOUS during the early floral meristem development; Regulates petal shape; Controls cell division during petal development; Acts through direct or indirect regulation of PHABULOSA and YAB1 and thus regulate cellular proliferation within the developing petal blade	Increased cell differentiation; Increased response to DNA damage; Increased defense response to most external biotic stimuli; Increased embryo development ending in seed dormancy; Increased gynoecium development; Decreased transcription by RNA polymerase II; Increased response to auxin; Increased response to cycloheximide; Increased response to hypoxia, oxidative stress, and excess silver ion	Sridhar, Vaniyambadi V., et al. "Transcriptional repression of target genes by LEUNIG and SEUSS, two interacting regulatory proteins for Arabidopsis flower development." Proceedings of the National Academy of Sciences 101.31 (2004): 11494-11499.
**Solyc06g084040.2**	**DRIP2**	**AT2G30580**	**0.32**	**E3 ubiquitin protein ligase DRIP2**	E3 ubiquitin-protein ligase that acts as a negative regulator of the response to water stress; Mediates ubiquitination and subsequent proteasomal degradation of the drought-induced transcriptional activator DREB2	Increased protein ubiquitination; Increased response to drought	Qin, Feng, et al. "Arabidopsis DREB2A-interacting proteins function as RING E3 ligases and negatively regulate plant drought stress–responsive gene expression." The plant cell 20.6 (2008): 1693-1707.
Solyc02g077320.2	SNI1	AT4G18470	0.32	SNI1 protein	Involved in DNA double-strand break repair; Negative regulator of hypersensitive response and systemic acquired resistance; Functions synergistically with NTL9/CBNAC as negative regulator of pathogen-induced PR1 expression; Suppresses defense response in the absence of pathogen challenge and is removed in response to induction	Increased response to DNA damage; Increased defense response to nematode; Increased histone H3 acetylation; Decreased defense response to pathogens; Decreased histone H3-K4 methylation; Decreased systemic acquired resistance; Increased regulation of transcription; Decreased hypersensitive response	Li, Xin, et al. "Identification and cloning of a negative regulator of systemic acquired resistance, SNI1, through a screen for suppressors of npr1-1." Cell 98.3 (1999): 329-339.
**Solyc11g010950.1**	**ELP4**	**AT3G11220**	**0.33**	**elongator complex protein 4**	Component of the RNA polymerase II elongator complex; Promotes organs development by modulating cell division rate; Regulates mechanisms producing carbon or importing sucrose; Involved in the repression of the abscisic acid signaling during seed germination; Required for auxin distribution or signaling; Prevents anthocyanins accumulation	Increased response to sucrose; Decreased anthocyanin metabolism; Increased cellular turnover; Increased auxin-mediated signaling; Increased regulation of carbon utilization; Increased regulation of leaf development; Increased response to oxidative stress; Increased tRNA wobble uridine modification	Nelissen, Hilde, et al. "The elongata mutants identify a functional Elongator complex in plants with a role in cell proliferation during organ growth." Proceedings of the National Academy of Sciences 102.21 (2005): 7754-7759.
**Solyc11g017300.1**	**CSN5A**	**AT1G22920**	**0.33**	**COP9 signalosome complex subunit 5a**	Protease subunit of the COP9 signalosome complex; Involved in photomorphogenesis and response to jasmonate; Essential regulator of the ubiquitin conjugation pathway; Involved in repression of photomorphogenesis in darkness; Required for degradation of PSIAA6	Decreased photomorphogenesis; Increased regulation of G2/M transition of mitotic cell cycle; Increased protein deneddylation; Increased red light phototransduction; Increased defense response to insects; Increased response to auxin; Increased floral organ development	Kwok, Shing F., et al. "Arabidopsis homologs of a c-Jun coactivator are present both in monomeric form and in the COP9 complex, and their abundance is differentially affected by the pleiotropic cop/det/fus mutations." The Plant Cell 10.11 (1998): 1779-1790.
Solyc04g082810.2	AHL27	AT1G20900	0.35	AT-hook motif nuclear-localized protein 27	Specifically binds AT-rich DNA sequences related to the nuclear matrix attachment regions; Negatively regulates plant innate immunity to pathogens through the down-regulation of PAMP-triggered FRK1 expression; Regulates flowering and hypocotyl elongation; Chromatin remodeling factor that negatively regulates leaf senescence	Increased chromatin organization; Increased flower development; Impaired innate immune response; Increased leaf senescence; Increased photomorphogenesis; Increased vegetative to reproductive phase transition of meristem	Lim, Pyung Ok, et al. "Overexpression of a chromatin architecture-controlling AT-hook protein extends leaf longevity and increases the post-harvest storage life of plants." The Plant Journal 52.6 (2007): 1140-1153.
**Solyc01g087170.2**	**N/a**	**AT2G41900**	**0.35**	**zinc finger CCCH domain-containing protein 30**	Involved in response to salt stress	Increased response to salt stress	Sun, Jiaqiang, et al. "The CCCH-type zinc finger proteins AtSZF1 and AtSZF2 regulate salt stress responses in Arabidopsis." Plant and Cell Physiology 48.8 (2007): 1148-1158.
**Solyc02g069310.2**	**NPR3**	**AT5G45110**	**0.36**	**regulatory protein NPR3**	Substrate-specific adapter of an E3 ubiquitin-protein ligase complex which mediates the ubiquitination and subsequent proteasomal degradation of target proteins; Regulates basal defense response to pathogens	Increased defense response to bacteria and fungus; Increased protein ubiquitination; Increased jasmonic acid mediated signaling; Increased systemic acquired resistance	Zhang, Yuelin, et al. "Negative regulation of defense responses in Arabidopsis by two NPR1 paralogs." The Plant Journal 48.5 (2006): 647-656.
**Solyc05g021100.2**	**SWAP70**	**AT2G30880**	**0.36**	**switch-associated protein 70**	Involved in intracellular signal transduction; Mediates defense response to bacteria	Increased defense response to bacteria; Increased intracellular signal transduction	Van Leeuwen, Wessel, et al. "Learning the lipid language of plant signalling." Trends in plant science 9.8 (2004): 378-384.
**Solyc07g005880.2**	**RFC1**	**AT5G22010**	**0.37**	**replication factor C subunit 1**	Broad regulator of transcriptional gene silencing, DNA replication, DNA repair, and the hypersensitive response; Required for DNA double-strand break repair and recombination; Important for lagging strand synthesis	Increased response to DNA damage; Increased DNA replication; Increased cellular turnover; Increased chromatin silencing; Increased H3-K9 methylation; Increased reproduction; Increased response to abscisic acid	Xia, S. T., et al. "Arabidopsis replication factor C subunit 1 plays an important role in embryogenesis." Zhi wu sheng li yu fen zi sheng wu xue xue bao= Journal of plant physiology and molecular biology 33.3 (2007): 179-187.
**Solyc01g096390.2**	**NRPE1**	**AT2G40030**	**0.37**	**DNA-directed RNA polymerase V subunit 1**	DNA-dependent RNA polymerase; Catalytic component of RNA polymerase V involved in RNA-directed DNA methylation-dependent silencing of endogenous repeated sequences; Essential component of siRNA production	Increased response to fungus; Increased DNA methylation; Increased posttranscriptional gene silencing; Increased transcription by RNA polymerase III	Pontier, Dominique, et al. "Reinforcement of silencing at transposons and highly repeated sequences requires the concerted action of two distinct RNA polymerases IV in Arabidopsis." Genes & development 19.17 (2005): 2030-2040.
**Solyc01g081330.2**	**ATJ1**	**AT1G28210**	**0.40**	**chaperone protein dnaJ 1, mitochondrial**	Plays a continuous role in plant development; Involved in the structural organization of cellular compartments under heat stress	Increased chaperone protein refolding; Increased response to heat	Park, Min Young, et al. "The Arabidopsis J protein AtJ1 is essential for seedling growth, flowering time control and ABA response." Plant and Cell Physiology 55.12 (2014): 2152-2163.
**Solyc02g021760.2**	**CPSF30**	**AT1G30460**	**0.40**	**30-kDa cleavage and polyadenylation specificity factor 30**	Component of the cleavage and polyadenylation specificity factor complex that plays a key role in pre-mRNA 3'-end formation and poly(A) addition; Involved in post-transcriptional control of oxidative stress responses; Regulates salicylic acid production	Increased mRNA polyadenylation; Increased hypersensitive response; Increased salicylic acid mediated signaling pathway; Increased response to oxidative stress; Increased RNA processing	Delaney, Kimberly J., et al. "Calmodulin interacts with and regulates the RNA-binding activity of an Arabidopsis polyadenylation factor subunit." Plant physiology 140.4 (2006): 1507-1521.
**Solyc08g082480.2**	**PI4KG4**	**AT2G46500**	**0.40**	**phosphatidylinositol 4-kinase gamma 4**	Phosphorylation of phosphatidylinositol to PI4P is the first committed step in the generation of phosphatidylinositol 4,5-bisphosphate	Increased regulation of flower development;Increased response to abscisic acid; Increased response to salt	Ma, Shisong, et al. "Loss of TIP1; 1 aquaporin in Arabidopsis leads to cell and plant death." The Plant Journal 40.6 (2004): 845-859.
**Solyc12g099010.1**	**GFS12**	**AT5G18525**	**0.40**	**protein GFS12**	Suppresses BCHC1, which is a negative regulator of storage vacuole trafficking and plant effector-triggered immunity	Increased defense response to bacteria; Increased protein targeting to vacuoles	Teh, Ooi-kock, et al. "BEACH-domain proteins act together in a cascade to mediate vacuolar protein trafficking and disease resistance in Arabidopsis." Molecular plant 8.3 (2015): 389-398.
**Solyc08g005270.2**	**RCD1**	**AT1G32230**	**0.41**	**inactive poly [ADP-ribose] polymerase RCD1**	Regulates hormonal responses during developmental; Required for embryogenesis, vegetative and reproductive development, and abiotic stress responses	Increased defense response to bacteria; Increased embryo development; Increased ethylene-activated signaling pathway; Increased jasmonic acid-mediated signaling; Increased lateral root morphogenesis; Increased programmed cell death; Increased response to drought, osmotic, ozone, and oxide stress	Ahlfors, Reetta, et al. "Arabidopsis RADICAL-INDUCED CELL DEATH1 belongs to the WWE protein–protein interaction domain protein family and modulates abscisic acid, ethylene, and methyl jasmonate responses." The Plant Cell 16.7 (2004): 1925-1937.
**Solyc01g111610.2**	**BRG3**	**AT3G12920**	**0.42**	**probable BOI-related E3 ubiquitin-protein ligase 3**	E3 ubiquitin-protein ligase	Increased defense response; Increased proteasome-mediated ubiquitin-dependent protein catabolic process; Increased programmed cell death	Park, Jeongmoo, et al. "DELLA proteins and their interacting RING Finger proteins repress gibberellin responses by binding to the promoters of a subset of gibberellin-responsive genes in Arabidopsis." The Plant Cell 25.3 (2013): 927-943.
**Solyc03g025940.1**	**N/a**	**AT3G48880**	**0.42**	**F-box/LRR-repeat protein**	Involved in endogenous messenger response to Gram-negative bacteria	Increased RNA signaling; Increased defense response to Gram-negative bacteria	Thieme, Christoph J., et al. "Endogenous Arabidopsis messenger RNAs transported to distant tissues." Nature Plants 1.4 (2015): 15025.
**Solyc03g121470.2**	**PLDALPHA4**	**AT1G55180**	**0.43**	**phospholipase D alpha 4**	Hydrolyzes glycerol-phospholipids at the terminal phosphodiesteric bond to generate phosphatidic acids; Promotes growth and plays a role in nitrogen signaling	Increased multidimensional cell division; Increased response to nitrogen, phosphate, and potassium starvation; Increased phospholipid catabolism; Increased nitrogen utilization; Increased post-embryonic development; Increased response to osmotic stress; Increased root development	Hong, Yueyun, et al. "Phospholipase Dε and phosphatidic acid enhance Arabidopsis nitrogen signaling and growth." The Plant Journal 58.3 (2009): 376-387.
**Solyc06g083510.2**	**PBL25**	**AT3G24790**	**0.44**	**serine/threonine-protein kinase PBL25**	Involved in protein phosphorylation signaling during germination and plant defense	Increased defense response; Increased protein phosphorylation; Increased reproduction	Wang, Yi, et al. "Transcriptome analyses show changes in gene expression to accompany pollen germination and tube growth in Arabidopsis." Plant physiology 148.3 (2008): 1201-1211.
**Solyc01g111600.2**	**HIPP26**	**AT4G38580**	**0.45**	**heavy metal-associated isoprenylated plant protein 26**	Heavy-metal-binding protein; Binds lead, cadmium and copper; Involved in heavy-metal transport; Involved in cadmium transport and play a role in cadmium detoxification	Increased acclimation during heat response; Increased metal ion transport; Increased response to cadmium stress	Gao, Wei, et al. "Arabidopsis thaliana acyl-CoA-binding protein ACBP2 interacts with heavy-metal-binding farnesylated protein AtFP6." New Phytologist 181.1 (2009): 89-102.
**Solyc05g052850.2**	**MYB1**	**AT3G09230**	**0.54**	**transcription factor MYB1**	Mediates salicylic acid signaling in response to salt stress	Increased response to salicylic acid; Increased response to salt stress	Wang, Ting, et al. "Salt-related MYB1 coordinates abscisic acid biosynthesis and signaling during salt stress in Arabidopsis." Plant physiology 169.2 (2015): 1027-1041.
**Solyc10g085000.1**	**BSK5**	**AT5G59010**	**0.55**	**serine/threonine-protein kinase BSK5**	Positive regulator of brassinosteroid signaling; Involved in abiotic stress tolerance; Required for abscisic acid-mediated response to drought and salt stress	Increased brassinosteroid-mediated signaling; Increased response to abscisic acid; Increased response to cold; Increased response to salt stress	Tang, Wenqiang, et al. "BSKs mediate signal transduction from the receptor kinase BRI1 in Arabidopsis." Science 321.5888 (2008): 557-560.
**Solyc02g077270.2**	**NCL**	**AT1G53210**	**0.66**	**sodium/calcium exchanger NCL1**	Participates in the maintenance of calcium homeostasis; Plays roles in auxin response, diurnal rhythm, and flowering time; Involved in response to salt stress	Improved calcium ion homeostasis; Increased calcium ion transmembrane transport; Increased response to salt stress	Wang, Peng, et al. "A Na+/Ca2+ exchanger-like protein (AtNCL) involved in salt stress in Arabidopsis." Journal of Biological Chemistry 287.53 (2012): 44062-44070.
**Solyc02g090490.2**	**PLP3**	**AT4G37050**	**0.70**	**patatin-like protein 3**	Catalyzes the hydrolysis of the neutral lipids monogalactosyldiacylglycerol, digalactosyldiacylglycerol, and phosphatidylglycerol; Plays a role in root development	Increased defense response; Increased lipid catabolism; Increased response to abscisic acid	Rietz, Steffen, et al. "Roles of Arabidopsis patatin-related phospholipases a in root development are related to auxin responses and phosphate deficiency." Molecular Plant 3.3 (2010): 524-538.
**Solyc11g069530.1**	**EDR2**	**AT4G19040**	**0.71**	**protein ENHANCED DISEASE RESISTANCE 2**	Negative regulator of the salicylic acid-mediated resistance to pathogen that limits initiation of cell death and the establishment of the hypersensitive response; Prevents ethylene-induced senescence	Increased ethylene-activated signaling pathway; Decreased leaf senescence; Increased hypersensitive response; Increased defense response to fungus; Increased response to ethylene; Increased response to salicylic acid	Tang, Dingzhong, et al. "Regulation of plant defense responses in Arabidopsis by EDR2, a PH and START domain-containing protein." The Plant Journal 44.2 (2005): 245-257.
**Solyc03g083350.2**	**PI4KG3**	**AT5G24240**	**0.72**	**phosphatidylinositol 4-kinase gamma 3**	Phosphorylation of phosphatidylinositol to PI4P is the first committed step in the generation of phosphatidylinositol 4,5-bisphosphate	Increased regulation of flower development; Increased response to abscisic acid; Increased response to salt	Ma, Shisong, et al. "Loss of TIP1; 1 aquaporin in Arabidopsis leads to cell and plant death." The Plant Journal 40.6 (2004): 845-859.
**Solyc01g096320.2**	**ATHB-12**	**AT3G61890**	**1.31**	**homeobox-leucine zipper protein ATHB-12**	Transcription activator that acts as growth regulators in response to drought	Increased development; Increased transcription; Increased response to abscisic acid; Increased response to virus; Increased response to drought and osmotic stress	Olsson, Anna, Peter Engström, and Eva Söderman. "The homeobox genes ATHB12 and ATHB7encode potential regulators of growth in response to water deficit in Arabidopsis." Plant molecular biology 55.5 (2004): 663-677.
**Solyc01g088520.2**	**DRP1E**	**AT3G60190**	**1.48**	**dynamin-related protein 1E**	Microtubule-associated force-producing protein of tubulo-vesicular network; Plays a major role in plasma membrane maintenance and cell wall integrity; Integral for plant growth and development	Increased cellular turnover; Increased response to fungus; Increased mitochondrial fission; Increased response to cadmium stress; Increased vesicle-mediated transport	Kang, Byung-Ho, et al. "The Arabidopsis cell plate-associated dynamin-like protein, ADL1Ap, is required for multiple stages of plant growth and development." Plant Physiology 126.1 (2001): 47-68.

**Table 2:** The 50 tomato plant DEGs associated with transcription and translation. DEGs were sorted by log2-fold change (log2FC). These DEGs were identified in the transcriptome analysis comparing psyllid-infested and uninfested tomato plants 3 weeks after infestation (*P* < 0.01). NCBI Blast searches were used to identify Gene IDs and protein products in tomatoes as well as their homologs in other species. Specifically, the expression changes in 44 genes (88%, **in bold**) would have resulted in improvements to transcription/translation pathways. These DEGs were related to post-translational modifications, miRNA processing, and gene silencing

Tomato gene ID	Gene ID	Homolog	Log2FC	Protein name	Uniprot description	Effect of psyllid infestation	Citation
Solyc01g087690.1	SIGD	AT5G13730	-1.06	RNA polymerase sigma factor sigD, chloroplastic	Sigma factors are initiation factors that promote the attachment of plastid-encoded RNA polymerase; Regulates transcription of the ndhF gene which codes for a subunit of the plastid NDH [NAD(P)H dehydrogenase] complex	Decreased response to light stimulus; Decreased transcription; Decreased regulation of RNA biosynthesis	Lerbs-Mache, Silva. "Function of plastid sigma factors in higher plants: regulation of gene expression or just preservation of constitutive transcription?." Plant molecular biology 76.3-5 (2011): 235-249.
Solyc06g073260.2	CSP41B	AT1G09340	-0.81	chloroplast stem-loop binding protein of 41 kDa b, chloroplastic	Associates with pre-ribosomal particles in chloroplasts and participates in chloroplast ribosomal RNA metabolism; Required for chloroplast integrity and embryo development; Regulates the circadian system; Regulates heteroglycans and monosaccharide mobilization	Impaired chloroplast organization; Impaired circadian rhythm; Decreased defense response to bacteria and wounding; Decreased galactose catabolism; Decreased monosaccharide metabolism; Decreased transcription and translation; Decreased response to cold and drought; Decreased rRNA processing	Raab, Sabine, et al. "ABA-responsive RNA-binding proteins are involved in chloroplast and stromule function in Arabidopsis seedlings." Planta 224.4 (2006): 900-914.
Solyc03g097320.2	SIGA	AT1G64860	-0.72	RNA polymerase sigma factor sigA	Essential protein that regulates psaA gene expression; Modulates photosystem stoichiometry; Maintains a harmonious electron flow and photosynthetic efficiency	Decreased response to light stimulus; Decreased cellular response to redox state; Decreased DNA-templated transcription; Impaired photosystem stoichiometry adjustment	Hakimi, Mohamed-Ali, et al. "Evolutionary conservation of C-terminal domains of primary sigma70-type transcription factors between plants and bacteria." Journal of Biological Chemistry 275.13 (2000): 9215-9221.
**Solyc05g055350.2**	**TRZ2**	**AT2G04530**	**-0.48**	**tRNase Z TRZ2, chloroplastic**	Zinc phosphodiesterase which displays tRNA 3'-processing endonuclease activity; Involved in tRNA maturation by removing a 3'-trailer from precursor tRNA	Decreased tRNA 3'-end processing	Schiffer, Steffen, Sylvia Rösch, and Anita Marchfelder. "Assigning a function to a conserved group of proteins: the tRNA 3′-processing enzymes." The EMBO journal 21.11 (2002): 2769-2777.
**Solyc11g066920.1**	**PCMP-H28**	**AT4G21065**	**-0.44**	**pentatricopeptide repeat-containing protein At4g21065**	Involved in RNA modification	Decreased RNA modification	Cheng, Chia-Yi, et al. "Araport11: a complete reannotation of the Arabidopsis thaliana reference genome." The Plant Journal 89.4 (2017): 789-804.
Solyc01g111020.2	MRL1	AT4G34830	-0.29	pentatricopeptide repeat-containing protein MRL1, chloroplastic	Regulator of the large subunit of RuBisCO; Involved in the processing and stabilization of the processed transcript	Decreased mRNA stabilization	Johnson, Xenie, et al. "MRL1, a conserved pentatricopeptide repeat protein, is required for stabilization of rbcL mRNA in Chlamydomonas and Arabidopsis." The Plant Cell 22.1 (2010): 234-248.
**Solyc03g034200.2**	**RCF3**	**AT5G53060**	**-0.26**	**RNA-binding KH domain-containing protein RCF3**	Negative regulator of osmotic stress-induced gene expression; Regulates thermotolerance responses under heat stress; Forms a complex with CPL1 that modulates co-transcriptional processes; Represses stress-inducible gene expression; Involved in primary miRNA processing and pre-miRNA biogenesis; Involved in JA-mediated fungal defense	Increased heat acclimation; Increased jasmonic acid-mediated signaling; Increased mRNA processing; Increased miRNA processing; Increased regulation of defense response to fungus; Increased regulation of gene expression; Increased response to osmotic stress; Increased RNA splicing	Xiong, Liming, et al. "HOS5–a negative regulator of osmotic stress-induced gene expression in Arabidopsis thaliana." The Plant Journal 19.5 (1999): 569-578.
**Solyc01g099300.1**	**MORC6**	**AT1G19100**	**0.25**	**protein MICRORCHIDIA 6**	Involved in RNA-directed DNA methylation during gene silencing; Regulates chromatin architecture/condensation to maintain gene silencing; Positive regulator of defense against oomycetes	Increased chromatin silencing; Increased fungal defense response; Increased DNA repair; Increased RNA-directed DNA methylation	Lorković, Zdravko J., et al. "Involvement of a GHKL ATPase in RNA-directed DNA methylation in Arabidopsis thaliana." Current Biology 22.10 (2012): 933-938.
**Solyc12g005330.1**	**RPL8A**	**AT2G18020**	**0.25**	**60S ribosomal protein L8-1**	Involved cytoplasmic translation	Increased cytoplasmic translation	Gordon, Sean P., et al. "Pattern formation during de novo assembly of the Arabidopsis shoot meristem." Development 134.19 (2007): 3539-3548.
**Solyc05g050200.1**	**ERF1A**	**AT4G17500**	**0.25**	**eukaryotic translation initiation factor 1A**	Required for maximal rate of protein biosynthesis; Enhances ribosome dissociation into subunits and stabilizes the binding of the initiator Met-tRNA(I) to 40 S ribosomal subunits	Increased formation of translation preinitiation complex; Increased translational fidelity	Li, Jigang, et al. "A subgroup of MYB transcription factor genes undergoes highly conserved alternative splicing in Arabidopsis and rice." Journal of experimental botany 57.6 (2006): 1263-1273.
**Solyc09g075640.1**	**FRS11**	**AT1G10240**	**0.25**	**FAR1-RELATED SEQUENCE 11**	Transcription activator involved in regulating light control of development	Increased regulation of transcription	Joly-Lopez, Zoé, et al. "Abiotic stress phenotypes are associated with conserved genes derived from transposable elements." Frontiers in Plant Science 8 (2017): 2027.
**Solyc04g056280.2**	**CDKC-1**	**AT5G10270**	**0.26**	**cyclin dependent kinase C-1**	Postranscriptional modifier; Involved in protein phosphorylation; Involved in leaf growth and development; Involved in defense reponse to virus	Increased leaf development; Increased phosphorylation of RNA polymerase II C-terminal domain; Increased defense response to virus	Pischke, Melissa S., et al. "A transcriptome-based characterization of habituation in plant tissue culture." Plant Physiology 140.4 (2006): 1255-1278.
**Solyc03g123640.2**	**APUM23**	**AT1G72320**	**0.26**	**pumilio homolog 23**	Sequence-specific RNA-binding protein that regulates translation and mRNA stability by binding the 3'-UTR of target mRNAs	Increased regulation of translation	Francischini, Carlos W., and Ronaldo B. Quaggio. "Molecular characterization of Arabidopsis thaliana PUF proteins–binding specificity and target candidates." The FEBS journal 276.19 (2009): 5456-5470.
**Solyc05g048850.2**	**RH8**	**AT4G00660**	**0.27**	**DEAD-box ATP-dependent RNA helicase 8**	ATP-dependent RNA helicase involved in mRNA turnover and mRNA decapping	Increased cytoplasmic mRNA processing body assembly and mRNA transport; Increased regulation of translation; Increased stress granule assembly; Increased viral process	Baek, Woonhee, et al. "A DEAD-box RNA helicase, RH8, is critical for regulation of ABA signalling and the drought stress response via inhibition of PP2CA activity." Plant, cell & environment 41.7 (2018): 1593-1604.
**Solyc05g051790.2**	**NRPB5A**	**AT3G22320**	**0.27**	**DNA-directed RNA polymerases II and IV subunit 5A**	Catalyzes the transcription of DNA into RNA; Component of RNA polymerase II which synthesizes mRNA precursors and many functional non-coding RNAs	Increased transcription by RNA polymerase I & II & II	Ream, Thomas S., et al. "Subunit compositions of the RNA-silencing enzymes Pol IV and Pol V reveal their origins as specialized forms of RNA polymerase II." Molecular cell 33.2 (2009): 192-203.
**Solyc11g005600.1**	**EIF2B**	**AT5G20920**	**0.28**	**eukaryotic translation initiation factor 2 subunit beta**	Functions in the early steps of protein synthesis; Binds to a 40S ribosomal subunit, followed by mRNA binding to form a 43S pre-initiation complex	Increased formation of cytoplasmic translation initiation complex; Increased formation of translation preinitiation complex	Ascencio-Ibáñez, José Trinidad, et al. "Global analysis of Arabidopsis gene expression uncovers a complex array of changes impacting pathogen response and cell cycle during geminivirus infection." Plant physiology 148.1 (2008): 436-454.
**Solyc06g008970.2**	**XPD**	**AT1G03190**	**0.28**	**general transcription and DNA repair factor IIH helicase subunit XPD**	Component of the general transcription and DNA repair factor IIH core comple; Plays an essential role in transcription initiation; Essential during plant growth; Negatively regulates a response to UV damage and heat stress	Increased DNA repair; Increased mitotic recombination; Increased transcription; Increased protein phosphorylation; Increased regulation of mitotic recombination; Increased response to heat, oxidative, and UV stress; Increased transcription by RNA polymerase II	Liu, Zongrang, et al. "Arabidopsis UVH6, a homolog of human XPD and yeast RAD3 DNA repair genes, functions in DNA repair and is essential for plant growth." Plant physiology 132.3 (2003): 1405-1414.
**Solyc01g096290.2**	**RPL40A**	**AT2G36170**	**0.28**	**ubiquitin-60S ribosomal protein L40**	Involved in protein degradation via the proteasome; Linear polymer chains formed via attachment by the initiator Met-lead during cellular signaling	Increased modification-dependent protein catabolism; Increased protein ubiquitination; Increased translation; Increased defense response to bacteria	Ditt, Renata F., et al. "The Arabidopsis thaliana transcriptome in response to Agrobacteria tumefaciens." Molecular plant-microbe interactions 19.6 (2006): 665-681.
**Solyc12g008450.1**	**N/a**	**AT2G40570**	**0.29**	**tRNA A64-2'-O-ribosylphosphate transferase**	Involved in charged-tRNA amino acid modification	Increased charged-tRNA amino acid modification	N/a
**Solyc04g082560.2**	**ITSN2**	**N/a**	**0.29**	**intersectin-2**	Adapter protein that provides indirect link between the endocytic membrane traffic and the actin assembly machinery; Regulates formation of clathrin-coated vesicles; Involved in endocytosis of integrin beta-1	Increased endocytosis; Increased dendrite extension; Increased regulation of Rho protein signal transduction; Increased viral process	Mettlen, Marcel, et al. "Endocytic accessory proteins are functionally distinguished by their differential effects on the maturation of clathrin-coated pits." Molecular biology of the cell 20.14 (2009): 3251-3260.
**Solyc08g082880.2**	**cox1101**	**N/a**	**0.29**	**rsm22-cox11 tandem protein 2, mitochondrial**	Involved in mitochondrion-encoded protein synthesis; Exerts its effect at some terminal stage of cytochrome c oxidase synthesis, probably by being involved in the insertion of the copper B into subunit I	Increased mitochondrial respiratory chain complex IV assembly; Increased mitochondrial translation	Khalimonchuk, Oleh, et al. "Sequential processing of a mitochondrial tandem protein: insights into protein import in Schizosaccharomyces pombe." Eukaryotic cell 5.7 (2006): 997-1006.
**Solyc06g062350.2**	**RIN1**	**AT5G22330**	**0.29**	**ruvB-like protein 1**	Core component of the chromatin remodeling INO80 complex which is involved in transcriptional regulation, DNA replication, and DNA repair; Component of the NuA4 histone acetyltransferase complex involved in transcriptional activation of select genes	Increased box C/D snoRNP assembly; Increased cell differentiation; Increased chromatin remodeling; Increased flower development; Increased meristem development; Increased regulation of defense response to fungus; Increased regulation of transcription by RNA polymerase II	Heyndrickx, Ken S., and Klaas Vandepoele. "Systematic identification of functional plant modules through the integration of complementary data sources." Plant physiology 159.3 (2012): 884-901.
**Solyc11g005130.1**	**UBN1**	**AT1G21610**	**0.30**	**ubinuclein-1**	Required for replication-independent chromatin assembly	Increased nucleosome organization; Increased regulation of gene silencing; Increased response to salt stress	N/a
**Solyc02g077320.2**	**SNI1**	**AT4G18470**	**0.32**	**SNI1 protein**	Involved in DNA double-strand break repair; Negative regulator of hypersensitive response and systemic acquired resistance; Functions synergistically with NTL9/CBNAC as negative regulator of pathogen-induced PR1 expression; Suppresses defense response in the absence of pathogen challenge and is removed in response to induction	Increased response to DNA damage; Increased defense response to nematode; Increased histone H3 acetylation; Decreased defense response to pathogens; Decreased histone H3-K4 methylation; Decreased systemic acquired resistance; Increased regulation of transcription; Decreased hypersensitive response	Li, Xin, et al. "Identification and cloning of a negative regulator of systemic acquired resistance, SNI1, through a screen for suppressors of npr1-1." Cell 98.3 (1999): 329-339.
**Solyc09g061340.1**	**PCMP-E76**	**AT2G13600**	**0.33**	**pentatricopeptide repeat-containing protein At2g13600**	Involved in mitochondrial mRNA modification during sugar metabolism	Increased mitochondrial mRNA modification; Increased RNA modification; Increased sugar-mediated signaling pathway; Increased sugar metabolism	Zhu, Qiang, et al. "SLO2, a mitochondrial pentatricopeptide repeat protein affecting several RNA editing sites, is required for energy metabolism." The Plant Journal 71.5 (2012): 836-849.
**Solyc08g076100.2**	**BZIP16**	**AT2G35530**	**0.33**	**bZIP transcription factor 16**	Transcriptional activator; G-box and G-box-like motifs are cis-acting elements defined in promoters of certain plant genes which are regulated by such diverse stimuli as light-induction or hormone control	Increased transcription; Increased intercellular signaling; increased photosynthesis; Increased plant growth	Shen, Huaishun, Kaiming Cao, and Xiping Wang. "AtbZIP16 and AtbZIP68, two new members of GBFs, can interact with other G group bZIPs in Arabidopsis thaliana." BMB reports 41.2 (2008): 132-138.
**Solyc10g074690.1**	**PABN1**	**AT5G51120**	**0.33**	**polyadenylate-binding protein 1**	Involved in the 3'-end formation of mRNA precursors	Increased mRNA processing	Cheng, Chia-Yi, et al. "Araport11: a complete reannotation of the Arabidopsis thaliana reference genome." The Plant Journal 89.4 (2017): 789-804.
**Solyc11g010950.1**	**ELP4**	**AT3G11220**	**0.33**	**elongator complex protein 4**	Component of the RNA polymerase II elongator complex; Promotes organs development by modulating cell division rate; Regulates mechanisms producing carbon or importing sucrose; Involved in the repression of the abscisic acid signaling during seed germination; Required for auxin distribution or signaling; Prevents anthocyanins accumulation	Increased response to sucrose; Decreased anthocyanin metabolism; Increased cellular turnover; Increased auxin-mediated signaling; Increased regulation of carbon utilization; Increased regulation of leaf development; Increased response to oxidative stress; Increased tRNA wobble uridine modification	Nelissen, Hilde, et al. "The elongata mutants identify a functional Elongator complex in plants with a role in cell proliferation during organ growth." Proceedings of the National Academy of Sciences 102.21 (2005): 7754-7759.
**Solyc05g007740.1**	**PCMP-H25**	**AT2G34370**	**0.33**	**pentatricopeptide repeat-containing protein At2g34370, mitochondrial**	Involved in RNA modification	Increased RNA modification	Guillaumot, Damien, et al. "Two interacting PPR proteins are major Arabidopsis editing factors in plastid and mitochondria." Proceedings of the National Academy of Sciences 114.33 (2017): 8877-8882.
Solyc08g007270.2	HAT4	AT4G16780	0.34	homeobox-leucine zipper protein HAT4	Negative regulator of cell elongation and proliferation; Mediator of the red/far-red light effects on leaf cell expansion under shade; Negatively regulates its own expression	Increased lateral root formation; Decreased regulation of transcription; Increased red light phototransduction; Increased response to auxin and cytokinin; Increased root development; Increased secondary thickening; Increased shade avoidance; Increased shoot system morphogenesis	Schena, Mark, Alan M. Lloyd, and Ronald W. Davis. "The HAT4 gene of Arabidopsis encodes a developmental regulator." Genes & development 7.3 (1993): 367-379.
**Solyc03g007100.2**	**CPSF160**	**AT5G51660**	**0.34**	**cleavage and polyadenylation specificity factor subunit 1**	Play sa key role in pre-mRNA 3'-end formation	Increased mRNA polyadenylation	Herr, Alan J., et al. "Defective RNA processing enhances RNA silencing and influences flowering of Arabidopsis." Proceedings of the National Academy of Sciences 103.41 (2006): 14994-15001.
**Solyc03g098420.2**	**PCMP-H37**	**AT2G01510**	**0.35**	**pentatricopeptide repeat-containing protein At2g01510**	Involved in RNA modification	Increased RNA modification	Cheng, Chia-Yi, et al. "Araport11: a complete reannotation of the Arabidopsis thaliana reference genome." The Plant Journal 89.4 (2017): 789-804.
**Solyc04g074910.2**	**RPS21B**	**AT3G53890**	**0.36**	**40S ribosomal protein S21-1**	Structural constituent of the ribosome	Increased chloroplast organization; Increased endonucleolytic cleavage to generate mature 3'-end of SSU-rRNA from (SSU-rRNA, 5.8S rRNA, LSU-rRNA); Increased translation	Wang, Ruijuan, et al. "Balance between cytosolic and chloroplast translation affects leaf variegation." Plant physiology 176.1 (2018): 804-818.
**Solyc05g005780.2**	**N/a**	**AT1G60070**	**0.37**	**AP-1 complex subunit gamma-2**	Subunit of clathrin-associated adaptor protein complex 1 that plays a role in protein sorting at the trans-Golgi network and early endosomes	Increased intracellular protein transport; Increased vesicle-mediated transport	Feng, Chong, et al. "Arabidopsis adaptor protein 1G is critical for pollen development." Journal of integrative plant biology 59.9 (2017): 594-599.
**Solyc06g076340.2**	**APUM2**	**AT2G29190**	**0.37**	**pumilio homolog 2**	Sequence-specific RNA-binding protein that regulates translation and mRNA stability by binding the 3'-UTR of target mRNAs	Increased regulation of translation	Francischini, Carlos W., and Ronaldo B. Quaggio. "Molecular characterization of Arabidopsis thaliana PUF proteins–binding specificity and target candidates." The FEBS journal 276.19 (2009): 5456-5470.
**Solyc02g078260.1**	**NRPB2**	**AT4G21710**	**0.37**	**DNA-directed RNA polymerase II subunit 2**	Catalyzes the transcription of DNA into RNA using the four ribonucleoside triphosphates as substrates; Contributes to the polymerase catalytic activity; Essential for the completion of mitosis in females	Increased production of miRNAs; Increased transcription by RNA polymerase II	Ream, Thomas S., et al. "Subunit compositions of the RNA-silencing enzymes Pol IV and Pol V reveal their origins as specialized forms of RNA polymerase II." Molecular cell 33.2 (2009): 192-203.
**Solyc01g096390.2**	**NRPE1**	**AT2G40030**	**0.37**	**DNA-directed RNA polymerase V subunit 1**	DNA-dependent RNA polymerase; Catalytic component of RNA polymerase V involved in RNA-directed DNA methylation-dependent silencing of endogenous repeated sequences; Essential component of siRNA production	Increased response to fungus; Increased DNA methylation; Increased posttranscriptional gene silencing; Increased transcription by RNA polymerase III	Pontier, Dominique, et al. "Reinforcement of silencing at transposons and highly repeated sequences requires the concerted action of two distinct RNA polymerases IV in Arabidopsis." Genes & development 19.17 (2005): 2030-2040.
**Solyc04g005690.2**	**N/a**	**AT1G14650**	**0.38**	**probable splicing factor 3A subunit 1**	Involved in mRNA splicing	Increased transcription by mRNA splicing	Dou, Kun, et al. "The PRP6-like splicing factor STA1 is involved in RNA-directed DNA methylation by facilitating the production of Pol V-dependent scaffold RNAs." Nucleic acids research 41.18 (2013): 8489-8502.
**Solyc07g049480.2**	**CPSF73-I**	**AT1G61010**	**0.39**	**cleavage and polyadenylation specificity factor subunit 3-I**	Play sa key role in pre-mRNA 3'-end formation	Increased mRNA 3'-end processing by stem-loop binding and cleavage; Increased mRNA polyadenylation; Increased snRNA processing	Herr, Alan J., et al. "Defective RNA processing enhances RNA silencing and influences flowering of Arabidopsis." Proceedings of the National Academy of Sciences 103.41 (2006): 14994-15001.
**Solyc05g047520.2**	**HEN2**	**AT2G06990**	**0.40**	**DExH-box ATP-dependent RNA helicase DExH10**	Involved in the degradation of a large number of non-coding nuclear exosome substrates; Involved in the maintenance of homeotic B and C gene expression in the reproductive whorl; Regulates floral organ spacing and identity	Increased maturation of 5.8S rRNA; Increased mRNA processing; Decreased posttranscriptional gene silencing; RNA catabolic process; Increased RNA metabolism; Increased floral organ development	Western, Tamara L., et al. "HUA ENHANCER2, a putative DExH-box RNA helicase, maintains homeotic B and C gene expression in Arabidopsis." Development 129.7 (2002): 1569-1581.
**Solyc02g021760.2**	**CPSF30**	**AT1G30460**	**0.40**	**30-kDa cleavage and polyadenylation specificity factor 30**	Component of the cleavage and polyadenylation specificity factor complex that plays a key role in pre-mRNA 3'-end formation and poly(A) addition; Involved in post-transcriptional control of oxidative stress responses; Regulates salicylic acid production	Increased mRNA polyadenylation; Increased hypersensitive response; Increased salicylic acid mediated signaling pathway; Increased response to oxidative stress; Increased RNA processing	Delaney, Kimberly J., et al. "Calmodulin interacts with and regulates the RNA-binding activity of an Arabidopsis polyadenylation factor subunit." Plant physiology 140.4 (2006): 1507-1521.
**Solyc12g049410.1**	**NUP107**	**AT3G14120**	**0.40**	**nuclear pore complex protein NUP107**	Involved in mRNA export from the nucleus by posttranscriptional tethering of RNA polymerase II; Involved in protein import into the nucleus	Increased mRNA transport from nucleus; Increased protein transort into nucleus	Parry, Geraint, et al. "The Arabidopsis SUPPRESSOR OF AUXIN RESISTANCE proteins are nucleoporins with an important role in hormone signaling and development." The Plant Cell 18.7 (2006): 1590-1603.
**Solyc03g025940.1**	**N/a**	**AT3G48880**	**0.42**	**F-box/LRR-repeat protein**	Involved in endogenous messenger response to Gram-negative bacteria	Increased RNA signaling; Increased defense response to Gram-negative bacteria	Thieme, Christoph J., et al. "Endogenous Arabidopsis messenger RNAs transported to distant tissues." Nature Plants 1.4 (2015): 15025.
**Solyc09g082520.2**	**RPS3AA**	**AT3G04840**	**0.47**	**40S ribosomal protein S3a-1**	Structural constituent of the ribosome	Increased translation	Chen, I-Peng, et al. "The transcriptional response of Arabidopsis to genotoxic stress–a high-density colony array study (HDCA)." The Plant Journal 35.6 (2003): 771-786.
**Solyc04g040170.1**	**NRPE5A**	**AT3G57080**	**0.49**	**DNA-directed RNA polymerase V subunit 5A**	Catalyzes the transcription of DNA into RNA; Component of RNA polymerase II which synthesizes mRNA precursors and many functional non-coding RNAs	Increased transcription by RNA polymerase I & II & III	Ream, Thomas S., et al. "Subunit compositions of the RNA-silencing enzymes Pol IV and Pol V reveal their origins as specialized forms of RNA polymerase II." Molecular cell 33.2 (2009): 192-203.
**Solyc05g032770.2**	**AL4**	**AT5G26210**	**0.53**	**PHD finger protein ALFIN-LIKE 4**	Histone-binding component that specifically recognizes H3 tails trimethylated on Lys-4	Increased chromatin organization; Increased regulation of transcription	Lee, Woo Yong, et al. "Arabidopsis ING and Alfin1-like protein families localize to the nucleus and bind to H3K4me3/2 via plant homeodomain fingers." The Plant Journal 58.3 (2009): 511-524.
**Solyc09g065850.2**	**AUX22**	**AT1G15580**	**0.64**	**auxin-induced protein AUX22**	Repressors of early auxin response genes at low auxin concentrations	Increased auxin-activated signaling; Increased regulation of transcription	Taniguchi, Masatoshi, et al. "Identification of gravitropic response indicator genes in Arabidopsis inflorescence stems." Plant signaling & behavior 9.9 (2014): e29570.
Solyc05g012210.2	AFP3	AT3G29575	0.76	ninja-family protein AFP3	Acts as a negative regulator of abscisic acid response and stress responses	Decreased transcription; Increased signal transduction	de Torres-Zabala, Marta, et al. "Pseudomonas syringae pv. tomato hijacks the Arabidopsis abscisic acid signalling pathway to cause disease." The EMBO journal 26.5 (2007): 1434-1443.
**Solyc08g007530.2**	**AHL1**	**AT4G12080**	**0.90**	**AT-hook motif nuclear-localized protein 1**	Specifically binds AT-rich DNA sequences related to the nuclear matrix attachment regions; Functions in the positioning of chromatin fibers within the nucleus	Increased transcription; Increased cellular turnover	Fujimoto, Satoru, et al. "Identification of a novel plant MAR DNA binding protein localized on chromosomal surfaces." Plant molecular biology 56.2 (2004): 225-239.
**Solyc01g096320.2**	**ATHB-12**	**AT3G61890**	**1.31**	**homeobox-leucine zipper protein ATHB-12**	Transcription activator that acts as growth regulators in response to drought	Increased development; Increased transcription; Increased response to abscisic acid; Increased response to virus; Increased response to drought and osmotic stress	Olsson, Anna, Peter Engström, and Eva Söderman. "The homeobox genes ATHB12 and ATHB7encode potential regulators of growth in response to water deficit in Arabidopsis." Plant molecular biology 55.5 (2004): 663-677.

**Table 3:** The 35 tomato plant DEGs associated with molecular signaling. DEGs were sorted by log2-fold change (log2FC). These DEGs were identified in the transcriptome analysis comparing psyllid-infested and uninfested tomato plants 3 weeks after infestation (*P* < 0.01). NCBI Blast searches were used to identify Gene IDs and protein products in tomatoes as well as their homologs in other species. Specifically, the expression changes in 28 genes (85%, **in bold**) would have resulted in improvements to molecular signaling pathways. These DEGs were related to protein phosphorylation and mobilization to the vacuole

Tomato gene ID	Gene ID	Homolog	Log2FC	Protein name	Uniprot description	Effect of psyllid infestation	Citation
Solyc08g083360.2	PNSB3	AT3G16250	-1.23	photosynthetic NDH subunit of subcomplex B 3, chloroplastic	NDH shuttles electrons from NAD(P)H:plastoquinone to quinones in the photosynthetic chain; Couples the redox reaction to proton translocation	Decreased photosynthetic electron transport	Qian, Haifeng, et al. "PGR5 and NDH pathways in photosynthetic cyclic electron transfer respond differently to sublethal treatment with photosystem-interfering herbicides." Journal of agricultural and food chemistry 62.18 (2014): 4083-4089.
Solyc01g087690.1	SIGD	AT5G13730	-1.06	RNA polymerase sigma factor sigD, chloroplastic	Promotes the attachment of plastid-encoded RNA polymerase; Regulates transcription of the ndhF gene	Decreased response to light stimulus; Decreased transcription; Decreased regulation of RNA biosynthesis	Lerbs-Mache, Silva. "Function of plastid sigma factors in higher plants: regulation of gene expression or just preservation of constitutive transcription?." Plant molecular biology 76.3-5 (2011): 235-249.
Solyc02g085950.2	RBCS3B	AT5G38410	-1.03	Ribulose bisphosphate carboxylase small chain	RuBisCO catalyzes two reactions: the carboxylation of D-ribulose 1,5-bisphosphate as well as the oxidative fragmentation of the pentose substrate; Both reactions occur simultaneously and in competition at the same active site	Decarbon fixation; Decreased chloroplast ribulose bisphosphate carboxylase complex assembly; Decreased photorespiration and photosynthesis; Decreaesed response to blue and red light	Menges, Margit, et al. "Cell cycle-regulated gene expression inArabidopsis." Journal of Biological Chemistry 277.44 (2002): 41987-42002.
Solyc12g036170.1	PNSB4	AT1G18730	-1.03	photosynthetic NDH subunit of subcomplex B 4, chloroplastic	NDH shuttles electrons from NAD(P)H:plastoquinone to quinones in the photosynthetic chain; Couples the redox reaction to proton translocation	Decreased photosynthetic electron transport	Qian, Haifeng, et al. "PGR5 and NDH pathways in photosynthetic cyclic electron transfer respond differently to sublethal treatment with photosystem-interfering herbicides." Journal of agricultural and food chemistry 62.18 (2014): 4083-4089.
Solyc11g006020.1	ndhO	AT1G74880	-0.93	NAD(P)H-quinone oxidoreductase subunit O, chloroplastic	NDH shuttles electrons from NAD(P)H:plastoquinone to quinones in the photosynthetic chain; Couples the redox reaction to proton translocation	Decreased NADH dehydrogenase complex assembly; Decreased photosynthesis	Ishikawa, Noriko, Tsuyoshi Endo, and Fumihiko Sato. "Electron transport activities of Arabidopsis thaliana mutants with impaired chloroplastic NAD (P) H dehydrogenase." Journal of plant research 121.5 (2008): 521-526.
Solyc02g066920.2	CRR7	AT5G39210	-0.84	protein CHLORORESPIRATORY REDUCTION 7, chloroplastic	Required for both formation and activity of the chloroplast NAD(P)H dehydrogenase complex of the photosynthetic electron transport chain; Required for the accumulation of NDH subcomplex A; Involved in post-translational steps during the biogenesis of subcomplex A	Decreased NAD(P)H dehydrogenase complex assembly	Kamruzzaman Munshi, M., Yoshichika Kobayashi, and Toshiharu Shikanai. "Identification of a novel protein, CRR7, required for the stabilization of the chloroplast NAD (P) H dehydrogenase complex in Arabidopsis." The Plant Journal 44.6 (2005): 1036-1044.
Solyc04g082930.1	LHCB7	AT1G76570	-0.82	chlorophyll a-b binding protein 7, chloroplastic	Captures and delivers excitation energy; Functions in non-photochemical quenching to dissipate energy; Contributes to primary photochemistry	Decreased photosynthesis and light harvesting in photosystem I; Impaired protein-chromophore linkage; Decreased response to blue and far-red light	Peterson, Richard B., and Neil P. Schultes. "Light-harvesting complex B7 shifts the irradiance response of photosynthetic light-harvesting regulation in leaves of Arabidopsis thaliana." Journal of plant physiology 171.3-4 (2014): 311-318.
Solyc10g077040.1	CRD1	AT3G56940	-0.80	magnesium-protoporphyrin monomethyl ester cyclase	Catalyzes the formation of the isocyclic ring in chlorophyll biosynthesis; Mediates the cyclase reaction	Decreased chlorophyll biosynthesis; Decreased chloroplast organization; Decreased photosynthesis; Decreased regulation of tetrapyrrole metabolic process	Tottey, Stephen, et al. "Arabidopsis CHL27, located in both envelope and thylakoid membranes, is required for the synthesis of protochlorophyllide." Proceedings of the National Academy of Sciences 100.26 (2003): 16119-16124.
Solyc06g048410.2	FSD1	AT4G25100	-0.79	iron superoxide dismutase [Fe] 1, chloroplastic	Breaks down superoxide anion radicals	Decreased response to cadmium stress; Decreased response to copper ion; Decreased response to light intensity; Decreased response to oxidative stress and ozone	Kuo, W. Y., et al. "CHAPERONIN 20 mediates iron superoxide dismutase (Fe SOD) activity independent of its co-chaperonin role in Arabidopsis chloroplasts." New Phytologist 197.1 (2013): 99-110.
Solyc02g080540.1	ATPC1	AT4G04640	-0.74	ATP synthase gamma chain, chloroplastic	Produces ATP from ADP in the presence of a proton gradient across the membrane	Decreased ATP biosynthesis; Decreased ATP synthesis coupled proton transport; Decreased photosynthetic electron transport in photosystem II	Dal Bosco, Cristina, et al. "Inactivation of the chloroplast ATP synthase γ subunit results in high non-photochemical fluorescence quenching and altered nuclear gene expression in Arabidopsis thaliana." Journal of Biological Chemistry 279.2 (2004): 1060-1069.
Solyc03g097320.2	SIGA	AT1G64860	-0.72	RNA polymerase sigma factor sigA	Essential protein; Controls the transcription of the psaA gene and thus modulates photosystem stoichiometry; Maintains a harmonious electron flow and photosynthetic efficiency	Decreased response to light stimulus; Decreased cellular response to redox state; Decreased DNA-templated transcription; Impaired photosystem stoichiometry adjustment; Decreased regulation of RNA biosynthesis	Hakimi, Mohamed-Ali, et al. "Evolutionary conservation of C-terminal domains of primary sigma70-type transcription factors between plants and bacteria." Journal of Biological Chemistry 275.13 (2000): 9215-9221.
Solyc07g032640.1	PSBO1	AT5G66570	-0.66	oxygen-evolving enhancer protein 1-1, chloroplastic	Stabilizes the manganese cluster which is the primary site of water splitting	Decreased defense response to bacteria; Decreased photoinhibition; Decreased photosynthesis; Decreased photosystem II assembly and stabilization; Decreased regulation of protein dephosphorylation	Murakami, Reiko, et al. "Characterization of an Arabidopsis thaliana mutant with impaired psbO, one of two genes encoding extrinsic 33-kDa proteins in photosystem II." FEBS letters 523.1-3 (2002): 138-142.
Solyc03g120430.2	GLYK	AT1G80380	-0.66	D-glycerate 3-kinase, chloroplastic	Indispensable ancillary metabolic pathway to the photosynthetic C3 cycle that enables land plants to grow in an oxygen-containing atmosphere	Impaired oxidative photosynthetic carbon pathway; Decreased photorespiration	Boldt, Ralf, et al. "D-GLYCERATE 3-KINASE, the last unknown enzyme in the photorespiratory cycle in Arabidopsis, belongs to a novel kinase family." The Plant Cell 17.8 (2005): 2413-2420.
Solyc08g080050.2	PGRL1A	AT4G22890	-0.64	PGR5 protein 1A, chloroplastic	Ferredoxin-plastoquinone reductase involved in cyclic electron flow around photosystem I	Decreased photosynthesis; Decreased photosynthetic electron transport in photosystem I	DalCorso, Giovanni, et al. "A complex containing PGRL1 and PGR5 is involved in the switch between linear and cyclic electron flow in Arabidopsis." Cell 132.2 (2008): 273-285.
Solyc10g007690.2	LHCA3	AT1G61520	-0.62	Photosystem I chlorophyll a/b-binding protein 3-1, chloroplastic	The light-harvesting complex functions as a light receptor; Captures and delivers excitation energy to photosystems with which it is closely associated	Decreased photosynthesis; Impaired protein-chromophore linkage; Decreased response to cold and light stress	Ganeteg, Ulrika, et al. "The properties of the chlorophyll a/b-binding proteins Lhca2 and Lhca3 studied in vivo using antisense inhibition." Plant physiology 127.1 (2001): 150-158.
Solyc01g005520.2	MET1	AT1G55480	-0.62	protein MET1, chloroplastic	Involved in photosystem II supercomplex formation and repair	Decreased photosynthesis	Ishikawa, Atsushi, et al. "Molecular characterization of the ZKT gene encoding a protein with PDZ, K-Box, and TPR motifs in Arabidopsis." Bioscience, biotechnology, and biochemistry 69.5 (2005): 972-978.
Solyc04g064670.2	PPD4	AT1G77090	-0.62	psbP domain-containing protein 4, chloroplastic	Involved in photosynthesis	Decreased photosynthesis	Dal Bosco, Cristina, et al. "Inactivation of the chloroplast ATP synthase γ subunit results in high non-photochemical fluorescence quenching and altered nuclear gene expression in Arabidopsis thaliana." Journal of Biological Chemistry 279.2 (2004): 1060-1069.
Solyc05g026550.2	ndhL	AT1G70760	-0.60	NAD(P)H-quinone oxidoreductase subunit L, chloroplastic	NDH shuttles electrons from NAD(P)H:plastoquinone to quinones in the photosynthetic chain; Couples the redox reaction to proton translocation	Decreased NADH dehydrogenase complex (plastoquinone) assembly; Decreased photosynthetic electron transport in photosystem I	Thieme, Christoph J., et al. "Endogenous Arabidopsis messenger RNAs transported to distant tissues." Nature Plants 1.4 (2015): 15025.
Solyc11g008620.1	PGLP1B	AT5G36790	-0.56	phosphoglycolate phosphatase 1B, chloroplastic	Photorespiratory enzyme that dephosphorylates the 2-phosphoglycolate produced by the RuBisCO oxygenation reaction	Decreased dephosphorylation; Decreased photorespiration	Reiland, Sonja, et al. "Large-scale Arabidopsis phosphoproteome profiling reveals novel chloroplast kinase substrates and phosphorylation networks." Plant physiology 150.2 (2009): 889-903.
Solyc04g057980.2	ndhH	ATCG01110	-0.56	NAD(P)H-quinone oxidoreductase subunit H, chloroplastic	NDH shuttles electrons from NAD(P)H:plastoquinone to quinones in the photosynthetic chain; Couples the redox reaction to proton translocation	Decreased photosynthesis; Decreased reaction to light	Lerbs-Mache, Silva. "Function of plastid sigma factors in higher plants: regulation of gene expression or just preservation of constitutive transcription?." Plant molecular biology 76.3-5 (2011): 235-249.
Solyc02g091560.2	SHM1	AT4G37930	-0.53	serine hydroxymethyltransferase 1, mitochondrial	Catalyzes interconversion of serine and glycine in the photorespiratory pathway; Involved in controlling cell damage caused by abiotic stress; Regulates the hypersensitive defense response	Decreased response to tetrahydrofolate; Decreased L-serine metabolism; Decreased one-carbon metabolism; Decreased photorespiration; Decreased hypersensitive response; Decreased response to cadmium, cold, heat, and light stress	Moreno, Juan Ignacio, et al. "Arabidopsis SHMT1, a serine hydroxymethyltransferase that functions in the photorespiratory pathway influences resistance to biotic and abiotic stress." The Plant Journal 41.3 (2005): 451-463.
Solyc05g052600.2	N/a	AT3G55800	-0.50	sedoheptulose-1,7-bisphosphatase, chloroplastic	Involved in fructose 1,6-biphosphate metabolism	Decreased defense response to bacteria; Decreased gluconeogenesis; Impaired reductive pentose-phosphate cycle; Decreased photosynthesis	Kiddle, Guy, et al. "Effects of leaf ascorbate content on defense and photosynthesis gene expression in Arabidopsis thaliana." Antioxidants and Redox Signaling 5.1 (2003): 23-32.
Solyc01g107660.2	SEP1	AT4G34190	-0.47	stress enhanced protein 1 protein	Involved in non-photochemical quenching; Plays a role in the thylakoid membrane in response to light stress	Decreased response to high light intensity; Decreased photosynthesis; Decreased response to wounding	Maejima, Kensaku, et al. "Degradation of class E MADS-domain transcription factors in Arabidopsis by a phytoplasmal effector, phyllogen." Plant signaling & behavior 10.8 (2015): e1042635.
Solyc10g080080.1	NTRC	AT2G41680	-0.46	NADPH-dependent thioredoxin reductase 3	Electron donor for plastidial 2-Cys peroxiredoxin; Required for chlorophyll biosynthesis and biogenesis of the photosynthetic apparatus; Regulates starch biosynthesis by redox activation of the ADP-glucose pyrophosphorylase	Impaired cell redox homeostasis; Decreased hydrogen peroxide catabolic process; Decreased regulation of chlorophyll biosynthesis; Decreased regulation of starch biosynthesis; Decreased removal of superoxide radicals	Moon, Jeong Chan, et al. "The C-type Arabidopsis thioredoxin reductase ANTR-C acts as an electron donor to 2-Cys peroxiredoxins in chloroplasts." Biochemical and biophysical research communications 348.2 (2006): 478-484.
Solyc02g072140.1	GIL1	AT5G58960	-0.44	protein GRAVITROPIC IN THE LIGHT 1	Required for red and far-red light-induced and phytochrome-mediated deregulation of negative gravitropism leading to randomization of hypocotyl growth orientation	Impaired gravitropism; Decreased response to red or far red light	Allen, Trudie, et al. "Phytochrome-mediated agravitropism in Arabidopsis hypocotyls requires GIL1 and confers a fitness advantage." The Plant Journal 46.4 (2006): 641-648.
Solyc11g008990.1	VIPP1	AT1G65260	-0.36	membrane-associated protein VIPP1, chloroplastic	Required for plastid vesicle formation and thylakoid membrane biogenesis	Decreased thylakoid membrane organization; Decreased vesicle organization	Kroll, Daniela, et al. "VIPP1, a nuclear gene of Arabidopsis thaliana essential for thylakoid membrane formation." Proceedings of the National Academy of Sciences 98.7 (2001): 4238-4242.
**Solyc05g014310.2**	**HHL1**	**AT1G67700**	**-0.34**	**protein HHL1, chloroplastic**	Involved in photoprotection; Forms a complex with LQY1 that is involved in the repair and reassembly cycle of the PSII-LHCII supercomplex under high-light conditions	Increased response to light stress	Jin, Honglei, et al. "HYPERSENSITIVE TO HIGH LIGHT1 interacts with LOW QUANTUM YIELD OF PHOTOSYSTEM II1 and functions in protection of photosystem II from photodamage in Arabidopsis." The Plant Cell 26.3 (2014): 1213-1229.
**Solyc05g050680.1**	**CKB4**	**AT2G44680**	**0.28**	**casein kinase II subunit beta-4**	Regulates the basal catalytic activity of the alpha subunit; Involved in the proteasome-dependent degradation of PIF1 and promotion of photomorphogenesis; Participates in the regulation of the initiation of translation	Improved circadian rhythm; Improved photoperiodism, Increased flowering	Dennis, Michael D., and Karen S. Browning. "Differential phosphorylation of plant translation initiation factors by Arabidopsis thaliana CK2 holoenzymes." Journal of Biological Chemistry 284.31 (2009): 20602-20614.
Solyc02g091410.2	DEGP7	AT3G03380	0.29	protease Do 7	Serine protease	Increased photoinhibition	Sun, Xuwu, et al. "The stromal chloroplast Deg7 protease participates in the repair of photosystem II after photoinhibition in Arabidopsis." Plant physiology 152.3 (2010): 1263-1273.
**Solyc05g055470.2**	**NAC078**	**AT5G04410**	**0.29**	**NAC domain-containing protein 78**	Transcriptional activated by transmembrane proteolysis; Induces flavonoid biosynthesis and required for the accumulation of anthocyanins in response to high light stress	Increased transcription; Increased regulation of flavonoid biosynthesis; Increased response to high light intensity	Morishita, Teruyuki, et al. "Arabidopsis NAC transcription factor, ANAC078, regulates flavonoid biosynthesis under high-light." Plant and Cell Physiology 50.12 (2009): 2210-2222.
**Solyc08g076100.2**	**BZIP16**	**AT2G35530**	**0.33**	**bZIP transcription factor 16**	Transcriptional activator; G-box and G-box-like motifs are cis-acting elements defined in promoters of certain plant genes	Increased transcription; Increased intercellular signaling; Increased photosynthesis; Increased plant growth	Shen, Huaishun, et al. "AtbZIP16 and AtbZIP68, two new members of GBFs, can interact with other G group bZIPs in Arabidopsis thaliana." BMB reports 41.2 (2008): 132-138.
Solyc11g017300.1	CSN5A	AT1G22920	0.33	COP9 signalosome complex subunit 5a	Involved in photomorphogenesis and response to jasmonate; Essential regulator of the ubiquitin conjugation pathway; Involved in repression of photomorphogenesis in darkness; Required for degradation of PSIAA6	Decreased photomorphogenesis; Increased cellular turnover; Increased protein deneddylation; Increased red light phototransduction; Increased defense response; Increased response to auxin; Increased floral organ development	Kwok, Shing F., et al. "Arabidopsis homologs of a c-Jun coactivator are present both in monomeric form and in the COP9 complex, and their abundance is differentially affected by the pleiotropic cop/det/fus mutations." The Plant Cell 10.11 (1998): 1779-1790.
**Solyc08g007270.2**	**HAT4**	**AT4G16780**	**0.34**	**homeobox-leucine zipper protein HAT4**	Negative regulator of cell elongation and specific cell proliferation processes ; Mediator of the red light response under light stress; Negatively regulates its own expression	Increased lateral root formation; Increased red light phototransduction; Increased response to auxin; Increased response to cytokinin; Increased secondary thickening; Increased shade avoidance	Schena, Mark, Alan M. Lloyd, and Ronald W. Davis. "The HAT4 gene of Arabidopsis encodes a developmental regulator." Genes & development 7.3 (1993): 367-379.
**Solyc04g082810.2**	**AHL27**	**AT1G20900**	**0.35**	**AT-hook motif nuclear-localized protein 27**	Negatively regulates innate immunity to pathogens through the down-regulation of PAMP-triggered FRK1 expression; Regulates flowering and hypocotyl elongation; Chromatin remodeling factor that negatively regulates leaf senescence	Increased chromatin organization; Increased flower development; Impaired innate immune response; Increased leaf senescence; Increased photomorphogenesis; Increased vegetative to reproductive phase transition of meristem	Lim, Pyung Ok, et al. "Overexpression of a chromatin architecture-controlling AT-hook protein extends leaf longevity and increases the post-harvest storage life of plants." The Plant Journal 52.6 (2007): 1140-1153.
**Solyc12g026400.1**	**DEGP9**	**AT5G40200**	**0.41**	**protease Do 9**	Serine protease that degrades the two-component response regulator ARR4; Acts upstream of ARR4 and regulates the activity of ARR4 in cytokinin and light-signaling pathways; Mediates the cross-talk between light and cytokinin signaling	Increased photosynthesis	Chi, Wei, et al. "DEG9, a serine protease, modulates cytokinin and light signaling by regulating the level of ARABIDOPSIS RESPONSE REGULATOR 4." Proceedings of the National Academy of Sciences 113.25 (2016): E3568-E3576.

**Table 4:** The 33 tomato plant DEGs associated with photosynthesis. DEGs were sorted by log2-fold change (log2FC). These DEGs were identified in the transcriptome analysis comparing psyllid-infested and uninfested tomato plants 3 weeks after infestation (*P* < 0.01). NCBI Blast searches were used to identify Gene IDs and protein products in tomatoes as well as their homologs in other species. Specifically, the expression changes in only 7 genes (20%, **in bold**) would have resulted in improvements to photosynthesis. Simultaneously, 28 DEGs, especially those related to response to light stimulus and photorespiration, underwent expression changes that would have resulted in impairments to photosynthesis

Tomato gene ID	Gene ID	Homolog	Log2FC	Protein name	Uniprot description	Effect of psyllid infestation	Citation
Solyc06g073260.2	CSP41B	AT1G09340	-0.81	chloroplast stem-loop binding protein of 41 kDa b, chloroplastic	Associates with pre-ribosomal particles in chloroplasts and participates in chloroplast ribosomal RNA metabolism; Required for chloroplast integrity and embryo development; Regulates the circadian system; Regulates heteroglycans and monosaccharide mobilization	Impaired chloroplast organization; Impaired circadian rhythm; Decreased defense response to bacteria and wounding; Decreased galactose catabolism; Decreased monosaccharide metabolism; Decreased transcription and translation; Decreased response to cold and drought; Decreased rRNA processing	Raab, Sabine, et al. "ABA-responsive RNA-binding proteins are involved in chloroplast and stromule function in Arabidopsis seedlings." Planta 224.4 (2006): 900-914.
Solyc11g042940.1	XK1	AT2G21370	-0.53	D-ribulose kinase precursor	Can phosphorylate D-ribulose with low efficiency	Decreased phosphorylation	Xie, Yuan, et al. "Crystal Structures of Putative Sugar Kinases from Synechococcus Elongatus PCC 7942 and Arabidopsis Thaliana." PloS one 11.5 (2016): e0156067.
Solyc02g069010.2	IMPL1	AT1G31190	-0.39	phosphatase IMPL1, chloroplastic	Phosphatase acting preferentially on D-myoinositol 1-phosphate	Decreased inositol biosynthesis; Decreased signal transduction	Torabinejad, Javad, et al. "VTC4 is a bifunctional enzyme that affects myoinositol and ascorbate biosynthesis in plants." Plant physiology 150.2 (2009): 951-961.
Solyc04g080860.1	LON2	AT5G47040	-0.37	lon protease 2	Mediates the selective degradation of misfolded and unassembled polypeptides in the peroxisomal matrix; Necessary for type 2 peroxisome targeting signal-containing protein processing	Decreased lateral root development; Decreased protein transport; Decreased protein processing; Decreased protein quality control for misfolded or incompletely synthesized proteins; Decreased protein targeting to peroxisome	Lingard, Matthew J., and Bonnie Bartel. "Arabidopsis LON2 is necessary for peroxisomal function and sustained matrix protein import." Plant physiology 151.3 (2009): 1354-1365.
Solyc05g006990.2	NPF4.6	AT1G69850	-0.29	protein NRT1/ PTR FAMILY 4.6	Low-affinity proton-dependent nitrate transporter; Involved in constitutive nitrate uptake; Involved in (+)-abscisic acid (ABA) transport; Mediates cellular ABA uptake	Decreased abscisic acid transport; Decreased nitrate assimilation; Decreased regulation of stomatal movement; Decreased response to nematode	Huang, Nien-Chen, et al. "Cloning and functional characterization of an Arabidopsis nitrate transporter gene that encodes a constitutive component of low-affinity uptake." The Plant Cell 11.8 (1999): 1381-1392.
**Solyc01g109520.2**	**RABG3F**	**AT3G18820**	**0.25**	**ras-related protein RABG3f**	Essential for trafficking from prevacuolar compartments; Essential for plant growth; Participates in the recruitment of the core retromer components to the endosomal membrane	Increased intracellular protein transport; Increased late endosome to vacuole transport; Increased Rab protein signal transduction; Increased vacuole organization	Zelazny, Enric, et al. "Mechanisms governing the endosomal membrane recruitment of the core retromer in Arabidopsis." Journal of Biological Chemistry 288.13 (2013): 8815-8825.
**Solyc09g074680.2**	**CUL1**	**AT4G02570**	**0.25**	**cullin-1**	Involved in ubiquitination and subsequent proteasomal degradation; Regulator of mitotic processes during gametogenesis and embryogenesis; Involved in floral organ development; Involved in auxin signaling; Regulates responses to jasmonates; Involved in phytochrome A light signaling; Involved in leaf senescence	Increased auxin-activated signaling pathway; Increased cellular cycling; Increased embryo development; Increased jasmonic acid-mediated signaling; Increased phloem or xylem histogenesis; Increased protein ubiquitination; Disrupted circadian rhythm; Increased response to jasmonic acid; Increased protein catabolism	Feng, Suhua, et al. "Arabidopsis CAND1, an unmodified CUL1-interacting protein, is involved in multiple developmental pathways controlled by ubiquitin/proteasome-mediated protein degradation." The Plant Cell 16.7 (2004): 1870-1882.
**Solyc03g034200.2**	**RCF3**	**AT5G53060**	**0.26**	**RNA-binding KH domain-containing protein RCF3**	Negative regulator of osmotic stress-induced gene expression; Regulates thermotolerance responses under heat stress; Forms a complex with CPL1 that modulates co-transcriptional processes; Represses stress-inducible gene expression; Involved in primary miRNA processing and pre-miRNA biogenesis; Involved in JA-mediated fungal defense	Increased heat acclimation; Increased jasmonic acid-mediated signaling; Increased mRNA processing; Increased miRNA processing; Increased regulation of defense response to fungus; Increased regulation of gene expression; Increased response to osmotic stress; Increased RNA splicing	Xiong, Liming, et al. "HOS5–a negative regulator of osmotic stress-induced gene expression in Arabidopsis thaliana." The Plant Journal 19.5 (1999): 569-578.
**Solyc12g095990.1**	**TIF4A-2**	**AT1G54270**	**0.26**	**eukaryotic initiation factor 4A-2**	Subunit of the eIF4F complex involved in cap recognition; Required for mRNA binding to ribosome; Unwinds RNA secondary structures in the 5'-UTR of mRNAs; Necessary for efficient binding of the small ribosomal subunit	Increased cytoplasmic translational initiation; Increased response to cadmium ion	Vergnolle, Chantal, et al. "The cold-induced early activation of phospholipase C and D pathways determines the response of two distinct clusters of genes in Arabidopsis cell suspensions." Plant physiology 139.3 (2005): 1217-1233.
**Solyc12g089150.1**	**SYP61**	**AT1G28490**	**0.26**	**syntaxin-61**	Vesicle trafficking protein that functions in the secretory pathway; Involved in osmotic stress tolerance and in abscisic acid regulation of stomatal responses	Increased abscisic acid-activated signaling; Increased Golgi vesicle transport; Increased intracellular protein transport; Increased vesicle docking and fusion	Shahriari, Mojgan, et al. "The AAA-type ATPase AtSKD1 contributes to vacuolar maintenance of Arabidopsis thaliana." The Plant Journal 64.1 (2010): 71-85.
**Solyc05g055600.2**	**VPS33**	**AT3G54860**	**0.27**	**vacuolar protein-sorting-associated protein 33 homolog**	Involved in regulating membrane fusion at the tonoplast and the prevacuolar compartment	Increased vesicle docking involved in exocytosis	Rojo, Enrique, et al. "The AtC–VPS protein complex is localized to the tonoplast and the prevacuolar compartment in Arabidopsis." Molecular biology of the cell 14.2 (2003): 361-369.
**Solyc08g065890.2**	**EPSIN1**	**AT5G11710**	**0.28**	**clathrin interactor EPSIN 1**	Plays a role in transport via clathrin-coated vesicles from the trans-Golgi network to endosomes; Stimulates clathrin assembly; Plays a role in the vacuolar trafficking of soluble cargo proteins at the trans-Golgi network	Increased protein targeting to vacuole	Song, Jinhee, et al. "Arabidopsis EPSIN1 plays an important role in vacuolar trafficking of soluble cargo proteins in plant cells via interactions with clathrin, AP-1, VTI11, and VSR1." The Plant Cell 18.9 (2006): 2258-2274.
**Solyc01g096290.2**	**RPL40A**	**AT2G36170**	**0.28**	**ubiquitin-60S ribosomal protein L40**	Involved in protein degradation via the proteasome; Linear polymer chains formed via attachment by the initiator Met-lead during cellular signaling	Increased modification-dependent protein catabolism; Increased protein ubiquitination; Increased translation; Increased defense response to bacteria	Ditt, Renata F., et al. "The Arabidopsis thaliana transcriptome in response to Agrobacteria tumefaciens." Molecular plant-microbe interactions 19.6 (2006): 665-681.
**Solyc08g007360.2**	**MAG5**	**AT5G47480**	**0.28**	**protein transport protein SEC16A homolog**	Required for efficient protein export from the endoplasmic reticulum to the Golgi; Functions as a scaffold and regulator of COPII coat assembly at ER exit sites	Increased COPII vesicle coating; Increased endoplasmic reticulum organization; Increased protein transport	Takagi, Junpei, et al. "MAIGO5 functions in protein export from Golgi-associated endoplasmic reticulum exit sites in Arabidopsis." The Plant Cell 25.11 (2013): 4658-4675.
**Solyc04g082560.2**	**ITSN2**	**N/a**	**0.29**	**intersectin-2**	Adapter protein that provides indirect link between the endocytic membrane traffic and the actin assembly machinery; Regulates formation of clathrin-coated vesicles; Involved in endocytosis of integrin beta-1	Increased endocytosis; Increased dendrite extension; Increased regulation of Rho protein signal transduction; Increased viral process	Mettlen, Marcel, et al. "Endocytic accessory proteins are functionally distinguished by their differential effects on the maturation of clathrin-coated pits." Molecular biology of the cell 20.14 (2009): 3251-3260.
**Solyc03g034180.2**	**GRF2**	**AT1G78300**	**0.31**	**14-3-3-like protein GF14 omega**	Associated with a DNA binding complex that binds to the G box	Increased brassinosteroid-mediated signaling	DeLille, Justin M.,et al. "The Arabidopsis 14-3-3 family of signaling regulators." Plant Physiology 126.1 (2001): 35-38.
**Solyc05g053830.2**	**N/a**	**AT4G14160**	**0.31**	**protein transport protein SEC23**	Promotes the formation of transport vesicles from the endoplasmic reticulum	Increased COPII-coated vesicle cargo loading; Increased intracellular protein transport	De Craene, Johan-Owen, et al. "Study of the plant COPII vesicle coat subunits by functional complementation of yeast Saccharomyces cerevisiae mutants." PLoS One 9.2 (2014): e90072.
**Solyc01g104970.2**	**BAK1**	**AT4G33430**	**0.31**	**brassinosteroid insensitive 1-associated receptor kinase 1**	Involved in brassinosteroid signaling response to beacteria/fungi/oomycetes; Mediates programmed cell death	Increased brassinosteroid mediated signaling pathway; Increased programmed cell death; Increased defense response to bacteria/fungus/oomycetes	Li, Jia, et al. "BAK1, an Arabidopsis LRR receptor-like protein kinase, interacts with BRI1 and modulates brassinosteroid signaling." Cell 110.2 (2002): 213-222.
**Solyc11g013260.1**	**PHB3**	**AT5G40770**	**0.32**	**prohibitin-3, mitochondrial**	Holdase/unfoldase involved in the stabilization of newly synthesized mitochondrial proteins; Necessary for mitochondrial and cell metabolism and biogenesis; Required to regulate ethylene-mediated signaling; Involved in growth maintenance; Functions in nitric oxide-mediated responses	Increased cellular turnover; Increased defense response to bacteria; Increased lateral root development; Increased mitochondrion organization; Increased response to auxin, ethylene, and nitric oxide; Increased to salt stress; Increased salicylic acid biosynthesis	Christians, Matthew J., et al. "Mutational loss of the prohibitin AtPHB3 results in an extreme constitutive ethylene response phenotype coupled with partial loss of ethylene-inducible gene expression in Arabidopsis seedlings." Journal of experimental botany 58.8 (2007): 2237-2248.
**Solyc09g061340.1**	**PCMP-E76**	**AT2G13600**	**0.33**	**pentatricopeptide repeat-containing protein At2g13600**	Involved in mitochondrial mRNA modification during sugar metabolism	Increased mitochondrial mRNA modification; Increased RNA modification; Increased sugar-mediated signaling pathway; Increased sugar metabolism	Zhu, Qiang, et al. "SLO2, a mitochondrial pentatricopeptide repeat protein affecting several RNA editing sites, is required for energy metabolism." The Plant Journal 71.5 (2012): 836-849.
**Solyc08g076100.2**	**BZIP16**	**AT2G35530**	**0.33**	**bZIP transcription factor 16**	Transcriptional activator; G-box and G-box-like motifs are cis-acting elements defined in promoters of certain plant genes which are regulated by such diverse stimuli as light-induction or hormone control	Increased transcription; Increased intercellular signaling; increased photosynthesis; Increased plant growth	Shen, Huaishun, et al. "AtbZIP16 and AtbZIP68, two new members of GBFs, can interact with other G group bZIPs in Arabidopsis thaliana." BMB reports 41.2 (2008): 132-138.
**Solyc11g010950.1**	**ELP4**	**AT3G11220**	**0.33**	**elongator complex protein 4**	Component of the RNA polymerase II elongator complex; Promotes organs development by modulating cell division rate; Regulates mechanisms producing carbon or importing sucrose; Involved in the repression of the abscisic acid signaling during seed germination; Required for auxin distribution or signaling; Prevents anthocyanins accumulation	Increased response to sucrose; Decreased anthocyanin metabolism; Increased cellular turnover; Increased auxin-mediated signaling; Increased regulation of carbon utilization; Increased regulation of leaf development; Increased response to oxidative stress; Increased tRNA wobble uridine modification	Nelissen, Hilde, et al. "The elongata mutants identify a functional Elongator complex in plants with a role in cell proliferation during organ growth." Proceedings of the National Academy of Sciences 102.21 (2005): 7754-7759.
**Solyc11g033270.1**	**M3KE1**	**AT3G13530**	**0.34**	**MAP3K epsilon protein kinase**	Serine/threonine-protein kinase involved in the spatial and temporal organization of cortical activity; Required for the normal functioning of the plasma membrane in developing pollen; Involved in the regulation of cell expansion, cell elongation, and embryo development	Increased cell division; Increased regulation of embryonic development; Increased regulation of cell growth; Increased signal transduction by protein phosphorylation	Seguí-Simarro, José M., et al. "Mitogen-activated protein kinases are developmentally regulated during stress-induced microspore embryogenesis in Brassica napus L." Histochemistry and cell biology 123.4-5 (2005): 541-551.
**Solyc01g096350.2**	**CRK3**	**AT2G46700**	**0.34**	**CDPK-related kinase 3**	Plays a role in signal transduction pathways that involve calcium as a second messenger; Serine/threonine kinase that phosphorylates histone H3 an GLN1-1	Increased response to abscisic acid stimulus; Increased intracellular signal transduction; Increased leaf senescence; Increased peptidyl-serine phosphorylation	Du, Wei, et al. "Biochemical and expression analysis of an Arabidopsis calcium-dependent protein kinase-related kinase." Plant science 168.5 (2005): 1181-1192.
**Solyc05g052510.2**	**CHC1**	**AT3G11130**	**0.34**	**clathrin heavy chain 1**	Clathrin is the major protein of the polyhedral coat of coated pits and vesicles; Mediates endocytosis and is required for a correct polar distribution of PIN auxin transporters	Increased clathrin-dependent endocytosis; Increased intracellular protein transport; Increased receptor-mediated endocytosis; Increased stomatal movement	Kitakura, Saeko, et al. "Clathrin mediates endocytosis and polar distribution of PIN auxin transporters in Arabidopsis." The Plant Cell 23.5 (2011): 1920-1931.
**Solyc02g030210.2**	**N/a**	**AT2G41710**	**0.36**	**AP2 ethylene-responsive transcription factor At2g41710**	Acts as a transcriptional activator; Binds to the GCC-box pathogenesis-related promoter element; Involved in the regulation of gene expression by stress factors and by components of stress signal transduction pathways	Increased ethylene-activated signaling pathway; Increased growth/development of reproductive tissues	Seki, Motoaki, et al. "Functional annotation of a full-length Arabidopsis cDNA collection." Science 296.5565 (2002): 141-145.
**Solyc02g069310.2**	**NPR3**	**AT5G45110**	**0.36**	**regulatory protein NPR3**	Substrate-specific adapter of an E3 ubiquitin-protein ligase complex; Mediates protein ubiquitination and subsequent proteasomal degradation; Regulates basal defense responses against pathogens	Increased defense response to bacteria and fungus; Increased protein ubiquitination; Increased jasmonic acid-mediated signaling; Increased systemic acquired resistance	Zhang, Yuelin, et al. "Negative regulation of defense responses in Arabidopsis by two NPR1 paralogs." The Plant Journal 48.5 (2006): 647-656.
**Solyc05g021100.2**	**SWAP70**	**AT2G30880**	**0.36**	**switch-associated protein 70**	Involved in intracellular signal transduction; Mediates defense response to bacteria	Increased defense response to bacteria; Increased intracellular signal transduction	Van Leeuwen, Wessel, et al. "Learning the lipid language of plant signalling." Trends in plant science 9.8 (2004): 378-384.
**Solyc01g089900.2**	**ALG12**	**AT1G02145**	**0.39**	**dol-P-Man:Man(7)GlcNAc(2)-PP-Dol alpha-1,6-mannosyltransferase**	Required for N-linked oligosaccharide assembly; Adds the eighth mannose residue in an alpha-1,6 linkage onto the dolichol-PP-oligosaccharide precursor dolichol-PP-Man7GlcNAc2	Increased dolichol-linked oligosaccharide biosynthesis; Increased N-linked glycosylation; Increased ERAD signaling	Hong, Zhi, et al. "Mutations of an α1, 6 mannosyltransferase inhibit endoplasmic reticulum–associated degradation of defective brassinosteroid receptors in Arabidopsis." The Plant Cell 21.12 (2009): 3792-3802.
**Solyc10g074570.1**	**CPK4**	**AT4G09570**	**0.39**	**calcium-dependent protein kinase 4**	Plays a role in signal transduction pathways that involve calcium as a second messenger; Regulator of the calcium-mediated abscisic acid signaling pathway	Increased intracellular signal transduction; Increased peptidyl-serine phosphorylation; Increased abscisic acid-activated signaling; Increased protein autophosphorylation	Rodriguez Milla, Miguel A., et al. "A novel yeast two-hybrid approach to identify CDPK substrates: Characterization of the interaction between AtCPK11 and AtDi19, a nuclear zinc finger protein1." FEBS letters 580.3 (2006): 904-911.
**Solyc12g099010.1**	**GFS12**	**AT5G18525**	**0.40**	**protein GFS12**	Acts predominantly to suppress BCHC1, which itself is a negative factor in protein storage vacuole trafficking regulation and plant effector triggered immunity	Increased defense response to bacteria; Increased protein targeting to vacuoles	Teh, Ooi-kock, et al. "BEACH-domain proteins act together in a cascade to mediate vacuolar protein trafficking and disease resistance in Arabidopsis." Molecular plant 8.3 (2015): 389-398.
**Solyc08g005270.2**	**RCD1**	**AT1G32230**	**0.41**	**inactive poly [ADP-ribose] polymerase RCD1**	Regulates hormonal responses during developmental; Required for embryogenesis, vegetative and reproductive development, and abiotic stress responses	Increased defense response to bacteria; Increased embryo development; Increased ethylene-activated signaling pathway; Increased jasmonic acid-mediated signaling; Increased lateral root morphogenesis; Increased nitric oxide biosynthesis; Increased programmed cell death; Increased response to drought, osmotic, ozone, and oxide stress	Ahlfors, Reetta, et al. "Arabidopsis RADICAL-INDUCED CELL DEATH1 belongs to the WWE protein–protein interaction domain protein family and modulates abscisic acid, ethylene, and methyl jasmonate responses." The Plant Cell 16.7 (2004): 1925-1937.
**Solyc03g025940.1**	**N/a**	**AT3G48880**	**0.42**	**F-box/LRR-repeat protein**	Involved in endogenous messenger response to Gram-negative bacteria	Increased RNA signaling; Increased defense response to Gram-negative bacteria	Thieme, Christoph J., et al. "Endogenous Arabidopsis messenger RNAs transported to distant tissues." Nature Plants 1.4 (2015): 15025.
**Solyc06g083510.2**	**PBL25**	**AT3G24790**	**0.44**	**serine/threonine-protein kinase PBL25**	Involved in protein phosphorylation signaling during germination and plant defense	Increased defense response; Increased protein phosphorylation; Increased reproduction	Wang, Yi, et al. "Transcriptome analyses show changes in gene expression to accompany pollen germination and tube growth in Arabidopsis." Plant physiology 148.3 (2008): 1201-1211.
**Solyc01g100720.2**	**IMPA4**	**AT1G09270**	**0.45**	**importin subunit alpha-4**	Mediates nuclear protein import across the nuclear envelope; Cellular receptor for the nuclear import of the virD2 protein of Agrobacteria	Increased defense response to symbiont of tumor, nodule or growth; Increased NLS-bearing protein transport into nucleus; Increased symbiont intracellular transport	Bhattacharjee, Saikat, et al. "IMPa-4, an Arabidopsis importin α isoform, is preferentially involved in Agrobacteria-mediated plant transformation." The Plant Cell 20.10 (2008): 2661-2680.
**Solyc10g085000.1**	**BSK5**	**AT5G59010**	**0.55**	**serine/threonine-protein kinase BSK5**	Positive regulator of brassinosteroid signaling; Involved in abiotic stress tolerance; Required for abscisic acid-mediated response to drought and salt stress	Increased brassinosteroid-mediated signaling; Increased response to abscisic acid; Increased response to cold; Increased response to salt stress	Tang, Wenqiang, et al. "BSKs mediate signal transduction from the receptor kinase BRI1 in Arabidopsis." Science 321.5888 (2008): 557-560.
**Solyc09g008460.2**	**RABC2A**	**AT5G03530**	**0.60**	**ras-related protein RABC2a**	Involved in intracellular vesicle trafficking and protein transport	Increased intracellular protein transport; Increased Rab protein signal transduction	Hashimoto, Kohsuke, et al. "An isoform of Arabidopsis myosin XI interacts with small GTPases in its C-terminal tail region." Journal of experimental botany 59.13 (2008): 3523-3531.
**Solyc05g012210.2**	**AFP3**	**AT3G29575**	**0.76**	**ninja-family protein AFP3**	Acts as a negative regulator of abscisic acid response and stress responses	Decreased transcription; Increased signal transduction	de Torres-Zabala, Marta, et al. "Pseudomonas syringae pv. tomato hijacks the Arabidopsis abscisic acid signalling pathway to cause disease." The EMBO journal 26.5 (2007): 1434-1443.

### Growth analysis

**Fig. 4:**
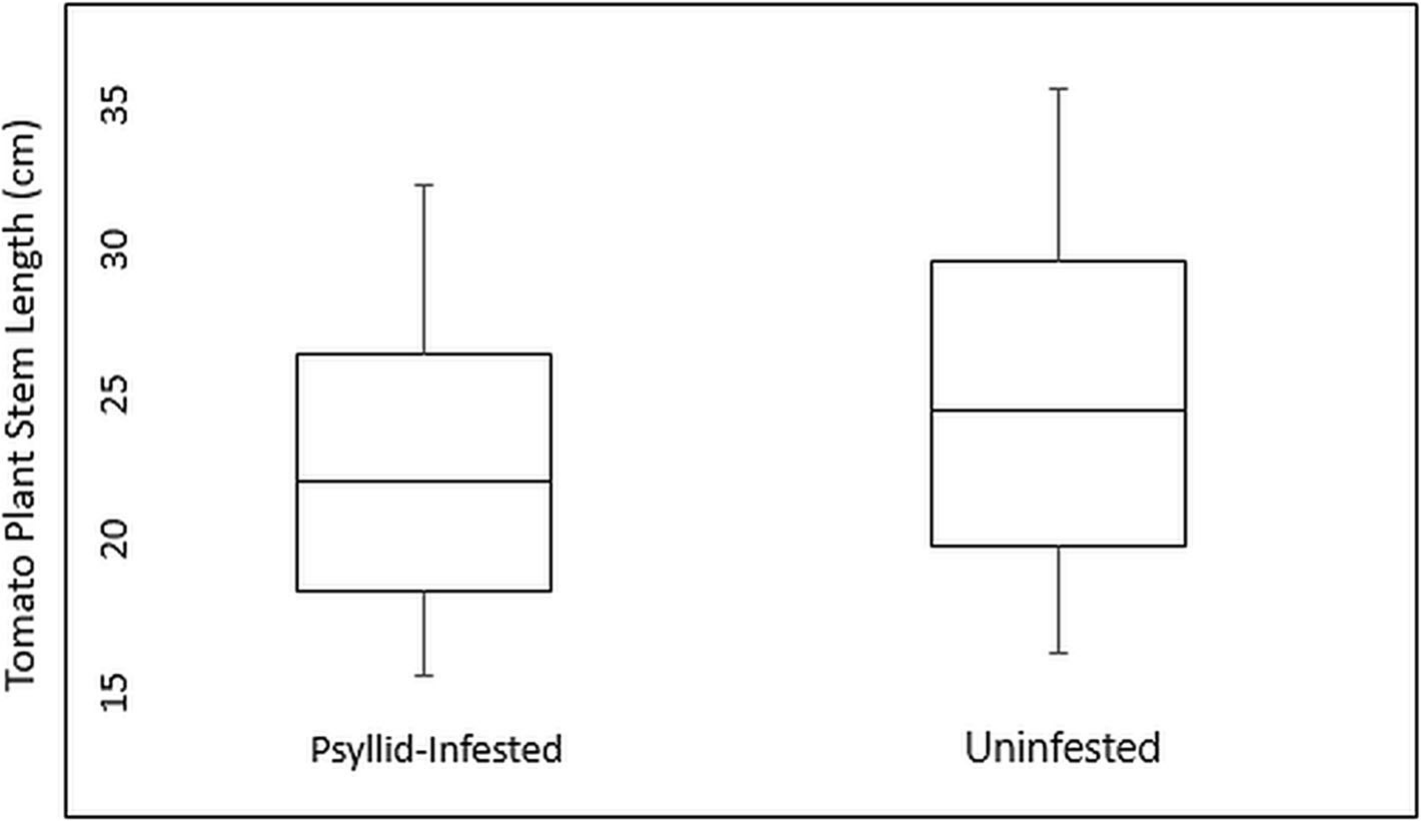
Boxplots of tomato stem length 3 weeks after psyllid-infested plants compared to uninfested plants. The ‘*’ denotes a significant difference based on a one-way Student’s t-Test for α = 0.05

The experiments tracking tomato stem growth rate showed, after 3 weeks, psyllid-infested plants (21.9 ± 0.8 cm, *n* = 28) were significantly shorter compared to uninfested plants (26.1 ± 0.7 cm, *n* = 27) (t-value = − 4.2, *P* < 0.001). These results suggested that psyllid infestation had lasting, negative consequences on tomato growth (Fig. 4).

### Psyllid development experiments

**Fig. 5:**
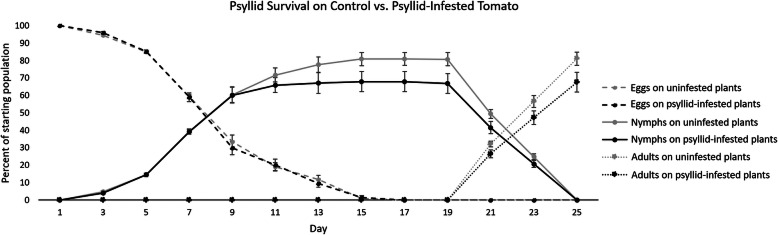
Development of a cohort of eggs and nymphs raised on previously psyllid-infested (black) and uninfested plants (grey). This graph reports the percentage number of eggs, nymphs, and adults present on the plants relative to the initial number of eggs laid on each plant

The psyllid development experiments showed that psyllids laid a statistically similar number of eggs on plants that had been previously infested (36.6 ± 13.4, *n* = 28) and uninfested plants (48.8 ± 12.1, *n* = 27) (t-score = − 0.71, *P* = 0.24). Also, the rate of egg hatching was similar between psyllids raised on previously infested plants (88.3 ± 6.7%) compared to psyllids raised on uninfested plants (89.1 ± 2.8%) (*n* = 55; t-score = 0.04, *P* = 0.48). In contrast, the same experiments showed that nymphs had a significantly lower survival rate when reared on previously psyllid-infested plants (71.9 ± 6.0%) compared to nymphs reared on uninfested plants (85.4 ± 3.7%) (t-score = − 1.89, *P* = 0.03). These differences, though, were only apparent after nymphs had spent 3–5 days on previously-psyllid infested plants. These results suggest that tomato plants responded to psyllid infestation by mounting an immune response that made them less suitable hosts for psyllid nymphs 3 weeks after the first infestation (Fig. 5).

## Discussion

Transcriptomic analysis of *S. lycopersicum* leaves showed that 362 genes were differentially expressed in tomato plants 3 weeks after psyllid infestation, suggesting that a week-long infestation by a small number of *B. cockerelli* had lasting consequences for gene expression in tomato plants (Figs. 1 and 2). Homologs of the DEGs were associated with 1) defense against abiotic and biotic stress, 2) transcription/translation, 3) molecular signaling, and 4) photosynthesis (Tables 1, 2, 3, 4; Supplementary Figure 3). In addition, RT-qPCR results corroborated the expression levels obtained by transcriptomic analysis for four tested genes (DRIP2, LON2, D27, PIP2–4) in the plants originally sequenced (Supplementary Figure 1) as well as plants independently grown and sampled (Supplementary Figure 2). Furthermore, the results of the tomato plant growth and psyllid development experiments were consistent with the results of the transcriptome analysis by demonstrating that psyllid infestation had lasting consequences for tomato plant growth (Fig. 4) and defense (Fig. 5). Specifically, the growth experiments demonstrated that tomato growth was stunted by psyllid infestation while the psyllid development experiments demonstrated that tomato plants that had been previously challenged by psyllids were less suitable hosts for nymphs.

Among the DEGs identified in the transcriptome analysis, 55 were homologs of genes associated with defense against biotic and abiotic stress (Table 1). For example, regulatory protein NPR3 (NPR3; Solyc02g069310.2) is a substrate-specific adapter of an E3 ubiquitin-protein ligase complex which mediates the ubiquitination and subsequent proteasomal degradation of target proteins, and consequently regulates the basal defense response to pathogens [69]. Since expression of NPR3 was significantly up-regulated (*P* = 0.001) in tomato plants 3 weeks after psyllid infestation, its associated defensive pathway was likely increased. Furthermore, NPR3 is involved in defense against insects, therefore its up-regulation may have been a consequence of plant defensive priming and/or the crosstalk between the jasmonic acid and salicylic acid pathways [16, 46]. Recently, a study performed in citrus plants showed that exposure to Asian citrus psyllids for 14 and 150 days resulted in induction of NPR1 and a delay in plant growth compared to the unfed plants. This effect was not detected after 7 days. The authors concluded that the prolonged exposure (~ 150 days) of citrus to Asian citrus psyllid feeding suppressed plant immunity and inhibited growth, probably through the salicylic acid signaling pathway [28]. Based on the functional characterization of *Arabidopsis* homologs, the expression changes observed in 80% stress-related DEGs would have likely coincided with increased responsiveness to abiotic and biotic stressors (see Table 1 for citations).

A subset of 50 DEGs were homologs of genes involved in transcription and/or translation (Table 2). For example, RNA-binding KH domain-containing protein RCF3 (RCF3; Solyc03g034200.2) is a negative regulator of osmotic stress-induced gene expression [67]. Since the expression of RCF3 was down regulated in tomato plants 3 weeks after psyllid infestation (*P* = 0.001), stress responsive gene expression would have increased. This interpretation is supported by the up regulation of genes such as homeobox-leucine zipper protein ATHB-12 (ATHB-12; Solyc01g096320.2), phospholipase D alpha 4 (PLDALPHA4; Solyc03g121470.2), and inactive poly [ADP-ribose] polymerase RCD1 (RCD1; Solyc08g005270.2). Furthermore, the expression profile changes observed in 88% of DEGs related to transcription/translation likely coincided with increased transcription/translation (see Table 2 for citations). Similarly, a subset of 35 genes were homologs of genes that function in molecular signaling (Table 3). In fact, the most common functional categories associated with DEGs were cellular processing and intracellular signaling (Fig. 3; Supplementary Figure 3). Together, these results suggest tomato plants were still active in responding to the psyllid threat 3 weeks after psyllids were last sensed by the plant.

A set of 33 DEGs were homologs of genes involved in photosynthesis (Table 4). For example, RNA polymerase sigma factor sigA (SIGA; Solyc03g097320.2) controls the transcription of the psaA gene and modulates photosystem stoichiometry, meaning its down regulation in tomato plants would have likely led to impaired photosynthesis after psyllid infestation [14, 19]. Furthermore, the expression changes in 26 (80%) DEGs related to photosynthesis would have likely also coincided with impaired photosynthesis. In support of this observation, the long-term, deleterious effects of psyllid infestation on tomato plant growth were evidenced by the experiments that tracked tomato plant stem length after psyllid infestation. These experiments showed the growth rate in tomato plant stems slowed after psyllid infestation (Fig. 4). These results were consistent with our previous study that observed stunted growth in tomato plants after psyllid infestation [40]. In addition to stunted stem growth, other developmental processes were likely impacted by psyllid infestation. For example, 6 DEGs were homologs of genes involved in auxin signaling. Since auxin-related signaling has several effects on plant growth and orientation, expression changes in these genes may be related to the stunting observed in tomato plants after psyllid infestation. Changes to plant growth, development, and photosynthesis post-herbivory may be related to the molecular crosstalk that takes place between plant defensive pathways and plant growth/development pathways [23, 26, 54].

Although 251 DEGs were homologs of genes for which published characterizations were available, 111 DEGs (30.7%) lacked any supporting information. This means nearly a third of the lasting consequences of psyllid infestation on tomato gene expression remain unknown. Of these DEGs, 78 (70.3%) were up-regulated in psyllid-infested plants relative to controls, consistent with the general pattern observed across DEGs. Therefore, it is reasonable to hypothesize that many of these expression changes would also be related to stress response, translation/transcription, molecular signaling, and/or photosynthesis.

In conclusion, the results of this manuscript are the first to report the long-lasting effects of psyllid herbivory on plant gene expression and health. The transcriptomic and growth experiments demonstrated that tomato plants underwent expression changes that likely repressed growth and developmental pathways in favor of promoting the expression of a select number of genes which are likely involved in defense against psyllid challenge. The DEGs that improved defense may constitute the genes directly involved in the tomato’s long-term response to psyllid challenge. This hypothesis is supported by the psyllid development experiments which showed psyllid nymphs had lower survival rates on psyllid-infested plants relative to uninfested plants (Fig. 5). The results presented in the current research showed that short exposures to small numbers of phloem feeding insects can have significant and lasting consequences for plant gene expression, growth, and defense. Alternatively, it is possible that the expression changes observed in tomato plants 3 weeks after psyllid infestation were a consequence of the accumulation of stress-related expression changes during psyllid infestation and sampling (with a razor blade). Continual stress can create negative feedback loops in stress-responsive genetic pathways [2]. This explanation is consistent with the overall deleterious impact of psyllid infestation observed in this study [9, 22]. Future disease biology research should continue exploring the long-term effects that vectors have on their hosts independent of their associated pathogens. These results should also be taken into consideration for epidemiologic studies of diseases associated with Liberibacter and their psyllid vectors.

## Methods

### Insect source

*B. cockerelli* were field-collected from Weslaco, Texas in 2008 and used to establish laboratory colonies. Tomato psyllid colonies have since been maintained on tomato plants under a 16: 8-h (Light: Dark) photoperiod at room temperature (22 ± 2 °C). The absence of Lso in these psyllid colonies was confirmed each month using the diagnostic PCR method previously described by Nachappa et al. [44]. Briefly, DNA from psyllids from the colony was extracted using the 10% CTAB method and subjected to PCR amplification of ‘*Candidatus* Liberibacter solanacearum’ 16S rDNA.

### Plant material

Tomato plants, cultivar Moneymaker (Victory Seed Company; Molalla, OR), were grown from seed in Metro-Mix 900 (Sun Gro Horticulture, Agawam, MA) soil and individually transplanted to 10 × 10 cm square pots 4 weeks later. Plants were watered every other day and fertilized weekly according to the manufacturer’s recommendation (Miracle-Gro® Water Soluble Tomato Plant Food; 18–18-21 NPK). All experiments were conducted at the same photoperiod (16: 8) and temperature (22 ± 2 °C) used to rear psyllids.

### Psyllid infestation and sample collection

Psyllid infestation were initiated when plants were 6 weeks old. Leaves branching below the apical meristem (i.e., leaves similar to the ones sampled for the transcriptome analysis) were caged with a small, white organza bag (amazon.com). Restricting psyllids to these leaves exposed them to systemic response of the plant to any prior infestation. Each bag either had no psyllids (control plants) or three adult male psyllids (psyllid-infested plants). Males were chosen to avoid the potentially confounding effect of oviposition on tomato gene expression. Seven days after infestation, caged tomato leaves were removed with a bleach-sterilized razor blade. Three weeks later, the top-most, fully developed leaf was sampled from each plant and immediately flash-frozen in liquid nitrogen. Samples were transferred to Eppendorf tubes and kept submerged under liquid nitrogen while ground with plastic, RNase-free pestles.

### RNA purification, sequencing and bioinformatic analysis

Total RNA extraction was performed on leaf tissue harvested 3 weeks after psyllid infestation using the Plant RNeasy Mini Kit (Qiagen, Valencia, CA) following the manufacturer’s protocol. Three biological replicates were sequenced per treatment (i.e., uninfested and psyllid-infested, six samples total). One fully-develop leaf and petiole were removed per biological replicate using sterilized razor blades. The top-most leaf was sampled to ensure that the gene expression changes observed were more likely to be associated with a plant systemic response. Samples were ground using sterilized plastic pestles. RNA samples were treated with RNase-Free DNase (Qiagen). Any remaining DNA was removed using the TURBO DNA-free™ Kit (Life Technologies, Carlsbad, CA). All remaining RNA was stored at − 80 °C for downstream quantitative reverse transcription PCR (RT-qPCR) validation. The isolated RNA was submitted to the Texas A&M Genomics and Bioinformatic Service for quality analysis, library preparation, and sequencing.

For transcriptomic sequencing, cDNA libraries were developed using the TruSeq RNA Library Prep Kit v2 (Illumina®; San Diego, CA) following the manufacturer’s protocol, generating 2 Х 150 bp read lengths. Libraries were multiplexed and sequenced on the Illumina PE HiSeq 2500 v4 platform. Sequence cluster identification, quality prefiltering, base calling, and uncertainty assessment were done in real time using Illumina’s HCS 2.2.38 and RTA 1.18.61 software with default parameter settings. Library preparation, sequencing, and read processing were performed by the Texas A&M Genomics and Bioinformatic Service. The processed sequences were uploaded to the CyVerse Discovery Environment computational infrastructure [17] where bioinformatic analysis was performed using the HISAT2-StringTie-Ballgown RNA-Seq workflow [31]. Libraries reads were mapped to the *S. lycopersicum* genome (vSL3.0) using HISAT2. StringTie assembled hits to known transcripts based on the vITAG3.2 annotation and made non-redundant with StringTie-Merge. DEGs were identified using Ballgown. Genes were considered differentially expressed when comparative q-values were below 0.01 [48]. DEG gene names were searched against the tomato genome database [15, 25] as well as the PhytoMine search engine in Phytozome [18]. DEGs were assigned putative functions based on their homology with other plant genes with known function published in Ensembl Plants (version SL2.50) and the UniProt Knowledgebase [6]. *Arabidopsis thaliana* homologs of DEGs were uploaded to the NCBI Gene Expression Omnibus (GEO) functional genomics data repository in order to visual overrepresentation among molecular pathways using the g:Profiler functional profiler.

### Transcriptome validation by RT-qPCR

To verify the results of the transcriptomic analysis, RT-qPCR analyses were performed on three genes differentially expressed in psyllid-infested plants: One putatively upregulated gene, an E3 ubiquitin-protein ligase that acts as a negative regulator of the response to water stress (Solyc06g084040.2 or DRIP2) [36] and two putatively downregulated genes, a peroxisomal protease potentially involved in drought stress response (Solyc04g080860.1 or LON2) and a chloroplastic Beta-carotene isomerase D27 (Solyc08g008630.2 or D27) [38, 65]. Since many of the regulatory genes differentially expressed in this study were involved in drought stress, an aquaporin (Solyc06g011350.2 or PIP2–4) that putatively underwent no regulatory change was selected as a control [29]. RT-qPCR experiments were conducted using RNA from the six sequenced tomato leaf samples (three per treatment) as well as six independently grown tomato plants (three per treatment), which were obtained by repeating the plant growth and infestation assays (three plants per treatment). This allowed for validation of the transcriptome results. An aliquot of 500 ng RNA was taken from each sample to develop cDNA libraries using the Verso™ cDNA Kit (Thermo Fisher Scientific, Waltham, MA), following the manufacturer’s manual. The cDNA libraries were diluted to 1:5 prior to RT-qPCR. Each reaction consisted of 1.0 μL cDNA, 5.0 μL SensiFAST SYBR Hi-ROX mix (Bioline, Memphis, TN), 0.4 μL of each primer (400 nM), and 3.6 μL of molecular grade water. Primers were designed using Primer3 [56], which targeted exons within a DEG, had an optimal annealing temperature of 60.0–62.0 °C, and generated 150 bp amplicons (Supplementary Table 1). RT-qPCR was performed in an Applied Biosystem QuantStudio 6 Flex system using the following parameters: 2 min at 95 °C, followed by 40 cycles of 5 s at 95 °C and 30 s at 60 °C. The melting curve for each reaction was generated to assure amplicon specificity. All RT-qPCR reactions were performed in triplicate. Relative expression levels for each gene were analyzed using the 2^-ΔΔCT^ method [51] with glyceraldehyde 3-phosphate dehydrogenase (GADPH) as a reference gene [27]. Since expression levels did not assume normality, they were analyzed using the Mann-Whitney U ranked test in JMP® Version 13 (SAS Institute Inc., Cary, NC, 1989–2018).

### Plant growth and psyllid development on previously infested and uninfested plants

Tomato plants were grown and treated using the same methods described above where 28 tomato plants were psyllid-infested and 27 plants were left uninfested. In order to minimize handling stress, plant growth was tracked using pictures taken 3 weeks after infestation to compare the total stem length of psyllid-infested plants to uninfested plants. Each picture included a 52 cm-long tray that served as a size standard. The total length (in pixels) of a tomato plant main stem was measured from the soil to the tip of the apical meristem using ImageJ1.X [58] and converted to centimeters using the length standard. This no-contact method of measurement was chosen to minimize plant wounding. Stem lengths were analyzed using a one-way student’s t-test in JMP.

Three weeks after initial infestation, three female psyllids were transferred to a no-choice cage and allowed to oviposit on undamaged leaves of the tomato plants that had previously been psyllid-infested or uninfested. As before, psyllids were restricted to a single leaf inside an organza bag, using a different leaf than the one used during the initial infestation. This exposed them to plant systemic conditions. Three adult females were caged together in each bag; there was one bag per plant. After 48 h, psyllids were removed, and their eggs were counted. Eggs were left on their respective plants and allowed to hatch. Nymphs were counted every other day and left to develop into adults. Adults were collected as they emerged. Egg hatching and nymph survival rates were calculated for the psyllids reared on each plant. Additionally, initial egg number and nymphal survival rates were compared between psyllids reared on previously infested and uninfested plants. Since 100% of the nymphs that survived development also emerged, adult emergence rate was not compared. Egg number and nymph survival were analyzed using student’s one-way t-tests in JMP.

## Supplementary Information

**Additional file 1: Supplementary Table 1.** Primer sequences used to target four specific genes for RT-qPCR experiments: One gene expressed at similar levels between control and psyllid-infested plants (PIP2–4, Solyc06g011350.2), one gene expressed at a higher level in psyllid-infested plants (DRIP2, Solyc06g084040.2), and two genes expressed at higher levels in uninfested plants (LON2, Solyc04g080860.1, and D27, Solyc08g008630.2). Asterisks indicate significant differences in expression.

**Additional file 2: Supplementary Table 2.** HISAT2 alignment summary of uninfested and psyllid-infested tomato plant transcriptomes to the *S. lycopersicum* vSL3.0 genome.

**Additional file 3: Supplementary Figure 1.** RT-qPCR results comparing ΔΔC_T_ values between control (white) and psyllid-infested (black) tomato plants. Samples were the same used for sequencing the tomato plant transcriptome. Tested genes were chosen based on the expected outcome predicted by the transcriptome analysis: One gene expressed at similar levels between uninfested and psyllid-infested plants (PIP2–4, Solyc06g011350.2), one gene expressed at a higher level in psyllid-infested plants (DRIP2, Solyc06g084040.2), and two genes expressed at higher levels in uninfested plants (LON2, Solyc04g080860.1, and D27, Solyc08g008630.2). Asterisks indicate significant differences in expression.

**Additional file 4: Supplementary Figure 2.** RT-qPCR results comparing ΔΔC_T_ values between control (white) and psyllid-infested (black) tomato plants. Samples were grown independent of the samples sequenced for the tomato plant transcriptome. Tested genes were chosen based on the expected outcome predicted by the transcriptome analysis: One gene expressed at similar levels between control and psyllid-infested plants (PIP2–4, Solyc06g011350.2), one gene expressed at a higher level in psyllid-infested plants (DRIP2, Solyc06g084040.2), and two genes expressed at higher levels in control plants (LON2, Solyc04g080860.1, and D27, Solyc08g008630.2). Asterisks indicate significant differences in expression.

**Additional file 5: Supplementary Figure 3.** Numerical results from the g:Profiler analysis. The first column depicts the ID of each circle from Fig. 3. The second column describes the GO information source (MF for molecular function, BP for ‘biological process’, and CC for ‘cellular component’) for each circle. The third column describes the term name associated with each circle. The fourth column describes the associated GO ID for the term. The fifth column shows the adjusted *p*-value for each term.

## Data Availability

Raw sequence data, processed data, and metadata were made available on the Gene Expression Omnibus (GEO) functional genomics repository under the ‘kharrison18’ directory (Accession # GSE165807). Other data including psyllid nymph counts, plant pictures, and RT-qPCR results can be obtained from the corresponding author, Dr. Kyle Harrison, upon request.

## Abbreviations

DEG(s): Differentially expressed gene(s)
fpkm: Fragments per kilobase per million reads
GEO: Gene Expression Omnibus
Lso: ‘*Candidatus* Liberibacter solanacearum’
NIFA: National Institute of Food and Agriculture
PCA: Principal component analysis
RT-qPCR: Quantitative reverse transcription PCR
