# Supplementary material for: Lasting consequences of psyllid (Bactericera cockerelli L.) infestation on tomato defense, gene expression, and growth
Source: BMC Plant Biol. 2021 Feb 24;21:114. doi: 10.1186/s12870-021-02876-z (PMC7905647; doi:10.1186/s12870-021-02876-z)
Supplement: Supplementary file 1 — Additional file 1: Supplementary Table 1. Primer sequences used to target four specific genes for RT-qPCR experiments: One gene expressed at similar levels between control and psyllid-infested plants (PIP2–4, Solyc06g011350.2), one gene expressed at a higher level in psyllid-infested plants (DRIP2, Solyc06g084040.2), and two genes expressed at higher levels in uninfested plants (LON2, Solyc04g080860.1, and D27, Solyc08g008630.2). Asterisks indicate significant differences in expression. [file 12870_2021_2876_MOESM1_ESM.pdf]

**Gene Name**	**Protein Name**	**Forward Primer (5'->3')**	**Reverse Primer (5'->3')**
Solyc04g080860.1	LON2	CGTTAAGCATGAGCGGCTGG	ATCATCCTCCCCATTCGCCG
Solyc06g011350.2	PIP2-4	AAATTTTATGCACTGACGTGACAA	CCACGCCTTGTCACCATTGA
Solyc06g084040.2	DRIP2	TGCAGGGATGGCAATGTTCC	AGTGCTAAATGACAGCCAAAGAGC
Solyc08g008630.2	D27	TACGGACAAGTACCACCGCC	TGGTGCCTGCGTAGATACAGA
