# Supplementary material for: Lasting consequences of psyllid (Bactericera cockerelli L.) infestation on tomato defense, gene expression, and growth
Source: BMC Plant Biol. 2021 Feb 24;21:114. doi: 10.1186/s12870-021-02876-z (PMC7905647; doi:10.1186/s12870-021-02876-z)
Supplement: Supplementary file 2 — Additional file 2: Supplementary Table 2. HISAT2 alignment summary of uninfested and psyllid-infested tomato plant transcriptomes to the S. lycopersicum vSL3.0 genome. [file 12870_2021_2876_MOESM2_ESM.pdf]

Treatment	Sample ID	Alignment Rate (%)	Reads
Control	1	96.31	17,358,465
Control	2	96.45	16,461,183
Control	3	95.99	18,455,303
Psyllid-infested	1	95.79	18,557,929
Psyllid-infested	2	96.64	17,347,190
Psyllid-infested	3	96.10	18,381,570
