# Supplementary figures and images for: Lasting consequences of psyllid (Bactericera cockerelli L.) infestation on tomato defense, gene expression, and growth
Source: BMC Plant Biol. 2021 Feb 24;21:114. doi: 10.1186/s12870-021-02876-z (PMC7905647; doi:10.1186/s12870-021-02876-z)

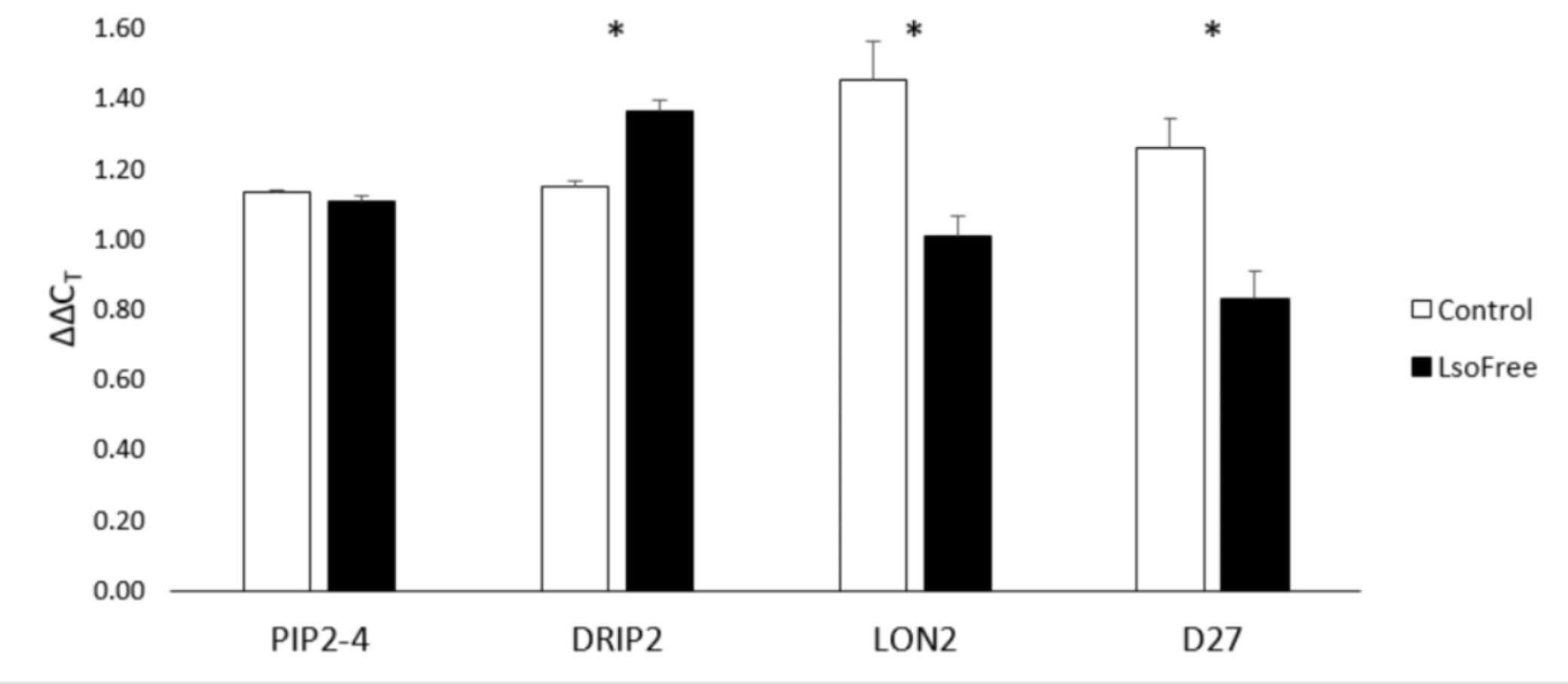

Supplement: Supplementary file 3 — Additional file 3: Supplementary Figure 1. RT-qPCR results comparing ΔΔCT values between control (white) and psyllid-infested (black) tomato plants. Samples were the same used for sequencing the tomato plant transcriptome. Tested genes were chosen based on the expected outcome predicted by the transcriptome analysis: One gene expressed at similar levels between uninfested and psyllid-infested plants (PIP2–4, Solyc06g011350.2), one gene expressed at a higher level in psyllid-infested plants (DRIP2, Solyc06g084040.2), and two genes expressed at higher levels in uninfested plants (LON2, Solyc04g080860.1, and D27, Solyc08g008630.2). Asterisks indicate significant differences in expression. [file 12870_2021_2876_MOESM3_ESM.tiff]

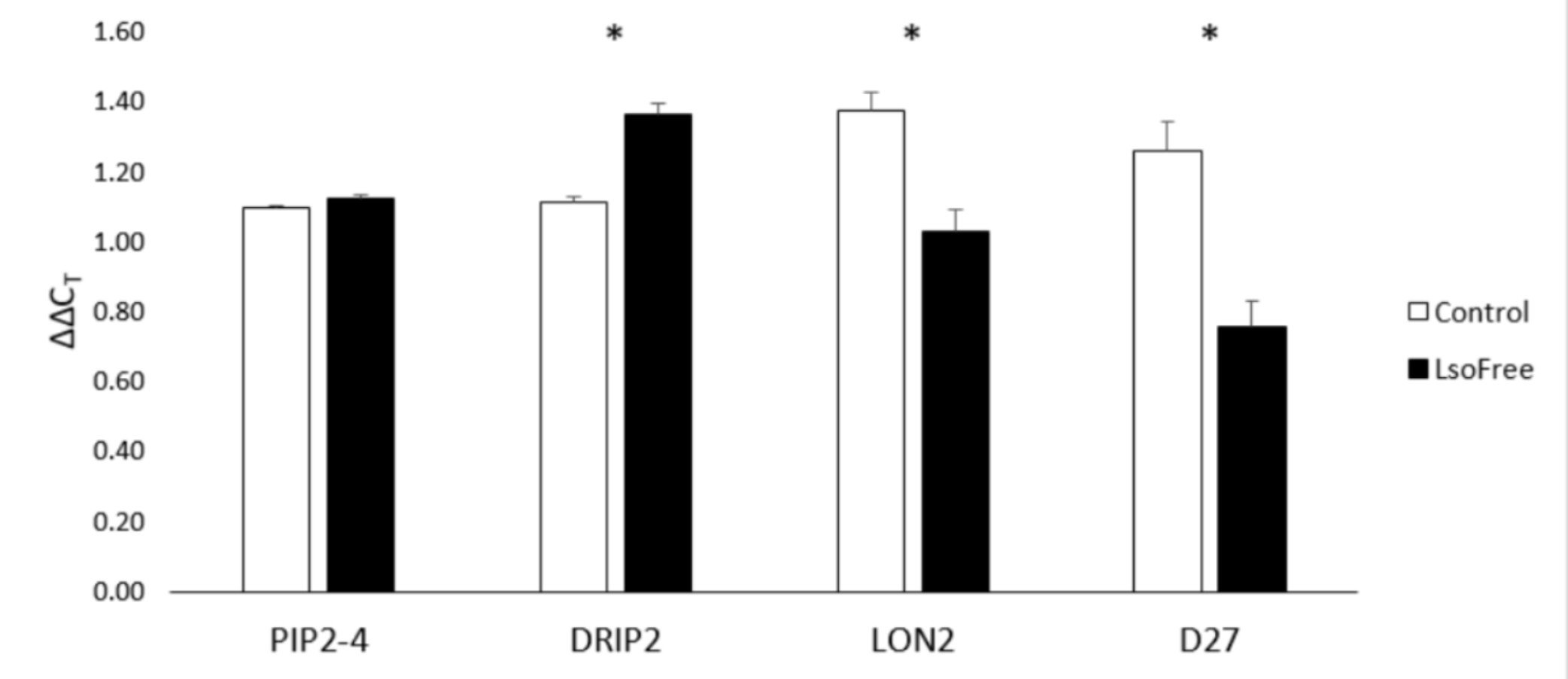

Supplement: Supplementary file 4 — Additional file 4: Supplementary Figure 2. RT-qPCR results comparing ΔΔCT values between control (white) and psyllid-infested (black) tomato plants. Samples were grown independent of the samples sequenced for the tomato plant transcriptome. Tested genes were chosen based on the expected outcome predicted by the transcriptome analysis: One gene expressed at similar levels between control and psyllid-infested plants (PIP2–4, Solyc06g011350.2), one gene expressed at a higher level in psyllid-infested plants (DRIP2, Solyc06g084040.2), and two genes expressed at higher levels in control plants (LON2, Solyc04g080860.1, and D27, Solyc08g008630.2). Asterisks indicate significant differences in expression. [file 12870_2021_2876_MOESM4_ESM.tiff]

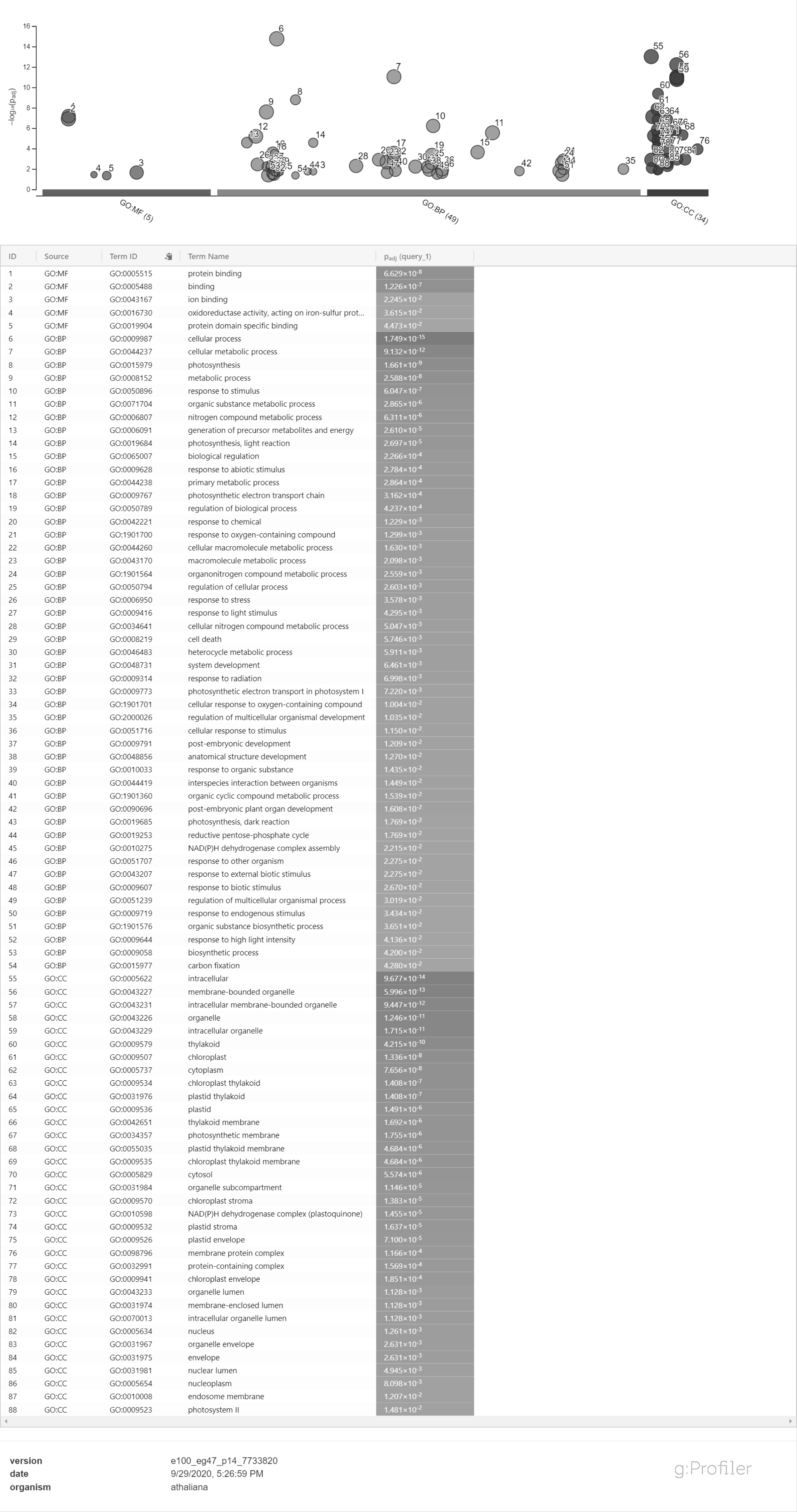

Supplement: Supplementary file 5 — Additional file 5: Supplementary Figure 3. Numerical results from the g:Profiler analysis. The first column depicts the ID of each circle from Fig. 3. The second column describes the GO information source (MF for molecular function, BP for ‘biological process’, and CC for ‘cellular component’) for each circle. The third column describes the term name associated with each circle. The fourth column describes the associated GO ID for the term. The fifth column shows the adjusted p-value for each term. [file 12870_2021_2876_MOESM5_ESM.tiff]
